# Supplementary material for: Tautomerism unveils a self-inhibition mechanism of crystallization
Source: Nat Commun. 2023 Feb 2;14:561. doi: 10.1038/s41467-023-35924-3 (PMC9893984; doi:10.1038/s41467-023-35924-3)
Supplement: Supplementary file 1 — Supplementary Information [file 41467_2023_35924_MOESM1_ESM.pdf]

# Supplementary Information for

### Tautomerism Unveils a Self-inhibition Mechanism of Crystallization

Weiwei Tang[1,2], Taimin Yang[3], Cristian A. Morales-Rivera[4], Xi Geng[1], Vijay K. Srirambhatla[5,6], Xiang Kang[2], Vraj P. Chauhan[1], Sungil Hong[4], Qing Tu[7], Alastair J. Florence[5,6], Huaping Mo[7], Hector A. Calderon[8,9], Christian Kisielowski[9], Francisco Robles Hernandez[10], Xiaodong Zou[3], Giannis Mpourmpakis[4], Jeffrey D. Rimer[1,*]

[1] University of Houston, Chemical and Biomolecular Engineering, Houston, TX 77204, USA
[2] Tianjin University, School of Chemical Engineering and Technology, State Key Laboratory of Chemical Engineering, The Co-Innovation Center of Chemistry and Chemical Engineering of Tianjin, Tianjin 300072, China
[3] Stockholm University, Department of Materials and Environmental Chemistry, SE-106 91, Stockholm, Sweden
[4] University of Pittsburgh, Chemical and Petroleum Engineering, Pittsburgh, PA 15261, USA
[5] EPSRC Future Manufacturing Research Hub for Continuous Manufacturing and Advanced Crystallization (CMAC) , University of Strathclyde, Glasgow, G1 1RD,Scotland, UK.Technology and Innovation Centre, 99 George Street, Glasgow, G1 1RD, UK
[6] Strathclyde Institute of Pharmaceutical and Biomedical Sciences, University of Strathclyde, Glasgow, G4 0RE, Scotland, UK
[7] Texas A&M University, Materials Science & Engineering, College Station, TX 77843, USA
[8] Purdue University, Medicinal Chemistry and Molecular Pharmacology, College of Pharmacy, West Lafayette, IN, USA
[9] Instituto Politecnico Nacional, ESFM-IPN, Departamento de Física, UPALM Zacatenco, CDMX 07338, Mexico
[10] The Molecular Foundry, Lawrence Berkeley National Laboratory, One Cyclotron Rd., Berkeley, CA 94720, USA
[11] University of Houston, Mechanical Engineering Technology, Houston, TX 77204, USA

**Supplementary materials:**

Supplementary Methods
Supplementary Discussion
Supplementary Figures 1 – 21
Supplementary Tables 1 – 5
Supplementary References (1 – 61)
Supplementary Movies 1 – 8

## 1. Supplementary Methods

**1.1 Materials**. The following chemicals were purchased from Sigma Aldrich (St Louis, MO): uric acid sodium salt (>98%), ammonium chloride (ACS reagent, ≥99.5%), uric acid anhydrous (≥99%), Guanine (>98%), allopurinol (>99%), ammonium hydroxide (28% $NH_3$ in $H_2O$, ≥99.99%), sodium hydroxide solution (1.0 N), hydrochloric acid (ACS reagent, 37%), uric acid-2-$^{13}$C,1,3,7-$^{15}N_3$ (≥98 atom%, ≥95% chemical purity), uric acid-1,3-$^{15}N_2$ (≥98 atom% $^{15}$N, ≥99% chemical purity), sodium chloride (BioXtra, ≥99.5%), 3-(trimethylsilyl)-1-propanesulfonic acid sodium salt (DSS sodium salt), and deuterium oxide ($D_2O$, 99.9 atom% D). These reagents were selected with high purity to limit the potential effects of impurities on ammonium urate crystallization. Deionized (DI) water used in all experiments was purified with an Aqua Solutions RODI water purification system (18.2 MΩ). All reagents were used as received. Filter paper (Grade 1, 11 μm pores) was purchased from Whatman, and Nylon syringe membrane (pore size 0.22 μm) was purchased from VWR.

**1.2 Preparation of crystal seeds for extended growth studies**
*Preparation of guanine crystal seeds.* A 20mM sodium guanine solution was prepared by dissolving appropriate guanine crystal powders in 1M NaOH aqueous solution at 60°C under rapid agitation. The clear solution was adjusted to be pH 7 using 30 wt% HCl aqueous solution for spontaneous crystallization. The obtained supernatant was then filtered through 0.22 μm filter membrane and transferred into a 3mL glass vial which was sealed with parafilm and left at ambient condition for 4-5 days to allow crystallization. The produced needle-like crystals were characterized by DXRxi Raman microscope (Thermo Fisher Scientific, USA) equipped with an EMCCD detector and a 532 nm laser source. Raman spectra were collected over the spectral range of 150-2000 cm$^{-1}$ at a spectral resolution of 3−5 cm$^{-1}$ under an exposure time of 10 s, confirmed to be anhydrous β form (Supplementary Fig. 16d).

*Preparation of allopurinol crystal seeds.* A 35mM sodium allopurinol solution was prepared by dissolving appropriate allopurinol powders in 1M NaOH aqueous solution at 80°C under rapid agitation. The clear solution was then adjusted to be pH 9.8 using 10 wt% HCl aqueous solution, filtered through 0.22 μm filter membrane, and transferred into a 110 mL glass bottle sealed with parafilm and left at ambient condition for about 3 days to allow crystallization. The crystalline phase of needle-like seed crystals was analyzed with a D/MAX 2500 X-ray diffractometer (XRD) using a Cu Kα source (40 kV, 100 mA) and was confirmed to be anhydrous form, the only stable crystal form reported so far, by a reference pattern from the solved crystal structure (*vide infra*). The crystal structure of allopurinol was solved by an Agilent-Rigaku Super Nova diffractometer with a CCD detector system (Supplementary Fig. 17d and Supplementary Table 4).

**1.3 Structure determination of $NH_4$HU crystals.** During 3D electron diffraction (3D ED) data collection, the position of the $NH_4$HU crystal during stage rotation was tracked by HAADF image stream using a previously reported method to track the crystal.[1] In total, 24 datasets were collected. The tilt range for each dataset was around 110°, the exposure time was 1 s/frame, and the rotation speed was around 0.6°/s. The electron dose rate was adjusted to 0.1e/Å$^2$/s and the total dose was around 18e/Å$^2$ for one dataset. The 3D reciprocal lattice was reconstructed using the REDp software[2] and the unit cell and space group were determined from the 3D reciprocal lattice. The possible space groups that match with the reflection conditions are *Cc* and *C*2/*c*. Data reduction and merging were performed using XDS.[3] Hierarchical analysis was performed using *EDtools*[4] and a dendrogram based on inter-correlation between all the collected datasets was generated for determining how to merge these datasets. In total, 10 datasets were merged. The merged dataset reached 96.7% in completeness and the average I/SIGMA reached 6.68. The structure of $NH_4$HU was solved by Shelxt.[5] During structure solution, all the non-hydrogen atoms were correctly identified by Shelxt with the space group *C2/c* while the solution with the space group *Cc* did not provide a chemically-sensible structure. The merged dataset was refined using Shelxl.[6] During structure refinement, the positions of hydrogen atoms were located one by one from the difference Fourier maps and Q-peaks and refined. All non-hydrogen atoms were refined anisotropically and hydrogen atoms on the urate

molecule were refined without applying any restraints (see Supplementary Table 1 for the statistics of data processing and refinement).

For indexing and structure solution processes, PXRD data were collected using a *2θ* scan range of 3–55°, step size of 0.017° *2θ*, and count time of 10 s/step. The final Rietveld refinement of PXRD data was performed on data collected using a variable counting time strategy. The powder pattern indexing was carried out using both DICVOL91/DICVOL04[7] as implemented in the DASH software (v.3.4.3)[8] and a singular value decomposition algorithm[9] implemented in TOPAS.[10] Pawley profile fitting[11] (Fig. 1h) confirmed that $NH_4HU$ crystallizes in the *C*2/*c* space group, with one urate and one ammonium in the asymmetric unit. The crystal structure of $NH_4HU$ was solved by the real-space approach[12] implemented in DASH. Lattice parameters, space group, molecular geometry of $NH_4HU$ crystals were all used as input for the structure solution procedure. The positions and orientations of the molecules in the unit cell were varied during the simulated annealing structure solution process. The best solution from the DASH runs was used in Rietveld refinement[13,14] in TOPAS. Standard restraints were applied to bond lengths, bond angles, and planarity. Rietveld refinement of the solved structure against the XRD data is shown in Supplementary Fig. 1d. The plot shows a good agreement between the experimental and calculated PXRD data ($R_{wp}$ = 6.13 %, $R_p$= 5.24 %). The final refined lattice parameters of $NH_4HU$ are a = 21.3906(13) Å, b = 3.53148(10) Å, c = 20.0868(10) Å, β=113.950(4) °, and V = 1386.72(12) $Å^3$. The crystal structure of $NH_4HU$ determined from powder X-ray diffraction matches closely with crystal structure determined using 3D ED, indicating that single crystal used in 3DED is representative of the bulk material.

**1.4 Microfluidic assays.** The growth rates of guanine and allopurinol crystals were measured in a microfluidic platform similar to that performed for $NH_4HU$. Growth solutions prepared with different concentrations (280-420 μM guanine or 6.0-7.1 mM allopurinol) at pH 7 were used in the microfluidics studies. The guanine aqueous solution was prepared by two steps: 0.1 M sodium guanine solution firstly prepared by dissolving appropriate amounts of guanine crystal powders in 0.4 M NaOH aqueous solution; and an appropriate volume of 0,1 M sodium guanine solution added into a 10 mL PBS buffer (pH 7.4) with pH adjustment (pH 7) by 30wt% HCl aqueous solution. The allopurinol aqueous solution was prepared by dissolving appropriate amounts of allopurinol powders in DI water at 60 ℃ under rapid agitation. The clear solution was cooled to room temperature and adjusted pH 7 by 10wt% HCl aqueous solution. The growth solution was filtered and then delivered to the device by a dual syringe pump (CHEMYX Fusion 4000) at a rate of 6 mL $h^{-1}$ for at least 240 min. Time-resolved optical micrographs of crystals (Supplementary Figs. 16b and 17b) were analyzed using Image J (NIH)[15] for quantifying incremental changes in crystal dimension along the *a*-direction. The growth rate was measured by linear regression of crystal size over time. The average growth rates were reported from measurements of at least 15 crystals. The flow rate of growth solution was adjusted to a sufficiently high value to circumvent the influence of diffusion and ensure growth rate measurements were performed in the kinetically-controlled regime.

**1.5 Analysis of crystal defects.** High-resolution TEM was performed by dose fractionation with low dose rates[16] at the ultimate detection limit where time series of lattice images are captured that allows monitoring any structure degradation with dose accumulation. A total dose (~ 100 $Å^{-2}$) could be accumulated before any detectable structure changes occurred by using a dose rate of 13 $eÅ^{-2}s^{-1}$ together with a resolution of 1.4 Å. All images were aligned and processed using the Tempas software package.[17] The image alignment is performed with sub-pixel accuracy. A lattice image that verifies the presence of dislocations in bent crystal areas is shown in Fig. 4f, where the presence of dislocations is highlighted by Fourier filtering.

**1.6 Atomic force microscopy (AFM).** The step velocity *v* was determined by measuring the displacements of 10 to 15 individual steps along the [010] direction with a step height h =0.87 ± 0.05 nm from time-resolved sequences of *in situ* AFM images captured in height mode. Between 5 and 15 measurements were taken for each individual step and the average growth rates were reported. The growth rate of advancing step bunches was measured in a similar way by monitoring the temporal change of individual step bunches. The average growth rates are reported from 5 to 10 individual step bunches and 10 to 15 measurements of

each step bunch. The rate of 2D nucleation, $J_{2D}$, was measured by counting the number of newly formed 2D islands on the surface scaled with the image area and time interval between successive images. The average $J_{2D}$ was obtained from 25 to 40 measurements at each condition tested.

According to the Gibbs-Thomson relation, $R_c = \Omega\alpha/\Delta\mu$, we evaluated the influence of minor tautomer inhibition on the interfacial energy of a step edge, α, by measuring the radius of the 2D nucleus of new layers. For these calculations, Ω is the molecular volume in the crystal and Δμ is the crystallization driving force defined in equation 20. The critical radius $R_c$ for layer nucleation is defined as the threshold size above which an island has a higher probability to grow and below which ($R < R_c$) islands are more likely to dissolve. We monitored the size evolution of all newly generated islands and classified them as either growing or dissolving. The largest sizes reached by dissolving islands and the threshold above which all islands grew were averaged to yield $R_c$. We used between 25 and 35 measurements to estimate the average $R_c$ at different urate concentrations and were plotted (Supplementary Fig. 18c) as a function of the supersaturation σ (see definition in equation 20).

AFM measurements for dissolution were performed in contact mode using a Cypher ES (Asylum Research, Santa Barbara, CA) and silicon nitride probes with a gold reflex coating. Samples were prepared on a glass slide and were attached to specimen disks (Ted Pella) covered with a thin layer of epoxy (Loctite, China). In situ experiments were conducted in ambient temperature by flowing DI water (pH 7) at 6 mL $h^{-1}$ into the liquid cell (ES-Cell-Gas) with a scan rate of 1.00 Hz at 256 lines per scan.

**1.7 Contact-resonance AFM (CR-AFM).** The CR-AFM cantilever dynamics has been well-established and summarized elsewhere.[18,19] Briefly, in CR-AFM, the cantilever beam can be approximated as a homogeneous, rectangular cantilever parallel to the surface. The flexural vibrations of the AFM cantilever can be described by solving the Euler-Bernoulli beam equation analytically:

$$E_t I \frac{\partial^4 y}{\partial x^4} + \rho A \frac{\partial^2 y}{\partial t^2} = 0 \qquad (1)$$

Here, $E_t$ is the modulus of elasticity of the tip, $\rho$ is the mass density, $A = ab$ denotes the cross section ($a$ is width and $b$ is height) and $I = \frac{ab^3}{12}$ describes the moment of inertia. The variables $x$ and $y$ denote the longitudinal coordinate and the deflection from the rest position, respectively. From equation (1), one can derive the dispersion relation

$$E_t I \alpha_n^4 - \rho A \omega_n^2 = 0 \qquad (2)$$

where $\omega_n$ is the angular frequency of the $n^{th}$ mode cantilever resonance and $\alpha_n$ is the wave number. The resonance frequencies are then given by[19]

$$f_n = \frac{(\alpha_n L)^2}{2\pi} \sqrt{\frac{E_t I}{\rho A L^4}} \qquad (3)$$

where $f_n$ is the free and/or contact resonance frequency of the cantilever. When the cantilever is in the free state, the characteristic equation[19]

$$1 + cos(\alpha_n L) cosh(\alpha_n L) = 0 \qquad (4)$$

can be used to calculate the wave number $\alpha_n$. The solutions to the characteristic equations are $\alpha_n L =$ 1.8751, 4.6941, 7.8547 for n = 1, 2, 3, respectively. When the cantilever is in contact with a stiff material (high Q value) and tip-sample vibrations are kept sufficiently small, the vertical damping can be neglected[20] and the characteristic equation becomes[19]

$$\frac{k^*}{k_c}\left\{\begin{array}{l}-\left[cosh(\alpha_n L_1)sin(\alpha_n L_1)-sinh(\alpha_n L_1)cos(\alpha_n L_1)\right]\times\left[1+cos(\alpha_n L')cosh(\alpha_n L')\right]\\+\left[cosh(\alpha_n L')sin(\alpha_n L')-sinh(\alpha_n L')cos(\alpha_n L')\right]\left[1-cos(\alpha_n L_1)cosh(\alpha_n L_1)\right]\end{array}\right\} \quad (5)$$

$$=2\frac{(\alpha_n L_1)^3}{3}\left[1+cos(\alpha_n L)cosh(\alpha_n L)\right]$$

where $k_c$ is the spring constant of the free cantilever and $L_1$ and L' are shown in Scheme 1. The relative tip position L'/L can be calibrated by the mode-equivalence method on a known sample[21,22] (here a silicon wafer is used, with E = 165 GPa, and $\upsilon$ = 0.22[23]), *i.e.*, measuring the contact resonance frequencies of the first two modes and solve for L'/L and $k^*/k_c$. The tip radius can then be calibrated by the Hertzian Contact model (Equation (1) in the paper). To extract the sample elastic modulus using equation (2) in the paper, the Poisson's ratio of the sample, $\upsilon_s$, is taken as 0.3, which is a typical value for crystalline materials.

**1.8 DFT calculations of tautomer interconversion.** The interconversion reaction between tautomer A ($[DKE\text{-}N_3]^-$) and tautomer B ($[DKE\text{-}N_9]^-$) in an aqueous medium at neutral pH can be described by the following reaction,

$$A + H_2O \xleftrightarrow{k_1,k_2} B + H_2O \quad (6)$$

where $k_1$ and $k_2$ are the rate constants for forward and reverse reactions. Assuming the excessive and constant concentration of $H_2O$ as 55.5 M, the interconversion reaction kinetics between diketo-enol tautomers can be approximately considered as a first-order reaction with rate $r_A$ expressed as

$$r_A = k[A] \quad (7)$$

where rate constant $k=(k_1+k_2)[H_2O]$. According to the Eyring equation[24], the rate constant $k_j$ (j=1, 2), assuming a transmission coefficient equal to 1, can be expressed by

$$k_1 = \frac{k_B T}{h}\left(c^\Theta\right)^{1-n}\exp\left(\frac{-\Delta G^\ddagger(A\rightarrow B)}{RT}\right) \quad (8)$$

$$k_2 = \frac{k_B T}{h}\left(c^\Theta\right)^{1-n}\exp\left(\frac{-\Delta G^\ddagger(B\rightarrow A)}{RT}\right) \quad (9)$$

where $k_B$ is Boltzmann's constant, $T$ is the absolute temperature, h = 6.626 x $10^{-34}$ $m^2\cdot kg/s$ is the Planck constant, $c^\Theta$ (= 1 M) is the standard concentration, n is the molecularity of the transition state (here n=2), R = 8.314 J/(K·mol) is the molar gas constant, $\Delta G^\ddagger$ (A→B) is the activation energy barrier for the forward reaction, and $\Delta G^\ddagger$ (B→A) is activation energy barrier for the reverse reaction. DFT calculations (M06-2X/6-311+G(d,p)) were used to compute the activation free energy barrier for the conversion from $[DKE\text{-}N_3]^-$ (tautomer A) to $[DKE\text{-}N_9]^-$ (tautomer B) as 73.3 kJ/mol and 70.7 kJ/mol for the forward and reverse reaction at 25°C, respectively (Supplementary Fig. 7a). This yields an overall rate constant $k$ = 192 $s^{-1}$ with half lifetime $t_{1/2}$ = 3.6 x $10^{-3}$ s, suggesting fast interconversion kinetics between the two diketo-enol tautomers in aqueous solution. We also evaluated the effect of temperature on rate constant and half lifetime (Supplementary Table 3). As expected, increasing temperature significantly speeds up the kinetics of tautomeric interconversion and thus explains the gradually sharpening of the carbonyl $^{13}C$ NMR peak (Fig. 3g).

## 2. Supplementary Discussion

### 2.1 Solution speciation and calculation of supersaturation

**2.1.1 Urate speciation and calculation of supersaturation.** The pH of crystallization media impacts the ionization speciation of urate. For calculations of the ionization equilibrium described herein, we treat $HU^-$ as the total available concentration of urate for $NH_4HU$ crystallization. The acid-base dissociation reactions of uric acid have two dissociation constants: $pKa_1 = 5.4$ and $pKa_2 = 10.3$ at room temperature.[25,26] The acid-base chemistry of urate involves two dissociations:

$$H_2U \rightarrow HU^- + H^+ \tag{10}$$

$$HU^- \rightarrow U^{2-} + H^+ \tag{11}$$

For each dissociation we use the following relationship to determine the relative concentrations of each acid (HA) and conjugate base ($A^-$):

$$pH = pK_a + \log\frac{[A^-]}{[HA]} \tag{12}$$

The sum of three urate species ($H_2U$, $HU^-$, and $U^{2-}$) is equal to the total concentration of urate, $C_{(urate)}$, which was measured experimentally by UV-Vis spectroscopy to account for potential loss during filtration steps:

$$C_{(urate)} = C_{(H_2U)} + C_{(HU^-)} + C_{(U^{2-})} \tag{13}$$

The concentration of urate species $HU^-$ in the $NH_4HU$ crystal structure is obtained by combining equations 12 and 13:

$$C_{(HU^-)} = \frac{C_{(urate)}}{1 + 10^{(pKa_1 - pH)} + 10^{(pH - pKa_2)}} \tag{14}$$

The concentration of ammonium cations is derived from electroneutrality (noting that $pK_W = 14$):

$$C_{(NH_4^+)} = C_{(HU^-)} + 2C_{(U^{2-})} + C_{(OH^-)} - C_{(H^+)} \tag{15}$$

$$C_{(U^{2-})} = C_{(HU^-)} 10^{(pH - pKa_2)} \tag{16}$$

$$C_{(OH^-)} = 10^{(pH - pKw)},\ C_{(H^+)} = 10^{-pH} \tag{17}$$

The concentration of each species $i$ is expressed as an activity ($a_i = \gamma_i \cdot C_i$) where the activity coefficient, $\gamma_i$, is calculated by the Davies equation [27]

$$\log\gamma_{(i)} = -0.5 z_{(i)}^2 \left( \frac{\sqrt{m}}{1 + \sqrt{m}} - 0.2m \right) \tag{18}$$

where $z_{(i)}$ is the ion charge of component i, and $m$ is the ionic strength ($m = 0.5\sum z_{(i)}^2\, C_{(i)}$).
At neutral pH 7, the predominant urate species is monovalent $HU^-$ whereas at high pH 11 the divalent $U^{2-}$ species is dominant (Supplementary Fig. 3a). The solubility product of $NH_4HU$ crystals, $K_{sp}$, is given by

$$K_{sp} = a_{e(HU^-)} a_{e(NH_4^+)} = C_{e(HU^-)} \gamma_{e(HU^-)} C_{e(NH_4^+)} \gamma_{e(NH_4^+)} \tag{19}$$

where $a_{e(i)}$ and $\gamma_{e(i)}$ are the equilibrium activity and activity coefficient of component i (i = $HU^-$ or $NH_4^+$), respectively. The term $C_{e(i)}$ is the equilibrium concentration of component i. At neutral pH, all experiments employ equimolar growth solutions (i.e. $C_{e(HU^-)} = C_{e(NH_4^+)}$). The solubility products $K_{sp}$ at pH 11 are 1.4 x $10^{-6}$ and 2.4 x $10^{-6}$ $mol^2 \cdot L^{-2}$ for temperatures used in microfluidics (21 °C) [26] and AFM (29 °C) experiments, respectively. The solubility products at pH 7 are 4.1 x $10^{-6}$ $mol^2 \cdot L^{-2}$ at 21 °C and 7.8 x $10^{-6}$ $mol^2 \cdot L^{-2}$ at 29 °C.

The supersaturation $\sigma$ of growth solution was calculated based on the following relationship:

$$\sigma = \frac{\Delta\mu}{k_B T} = \frac{1}{2}\ln\left(\frac{a_{(U^-)}a_{(NH_4^+)}}{K_{sp}}\right) = \frac{1}{2}\ln\left(\frac{C_{(U^-)}\gamma_{(U^-)}C_{(NH_4^+)}\gamma_{(NH_4^+)}}{K_{sp}}\right) \quad (20)$$

where $\Delta\mu$ is chemical potential between supersaturated growth solution and equilibrium saturated solution, i.e. crystallization driving force. For conditions selected in the manuscript for microfluidics and AFM measurements, the supersaturations are within a narrow range owing to similar trends in $C_{(HU^-)}$ and $K_{sp}$. For example, using a fixed urate concentration of 12 mM results in $\sigma$=1.21 at pH 7 and $\sigma$=1.15 at pH 11.

**2.1.2 Guanine and allopurinol speciation and supersaturation calculation.** The solution speciations of guanine and allopurinol were derived from ionization equilibrium and mass balance using the same method as that for urate speciation. The acid-base dissociation reactions of guanine have three dissociation constants: $pKa_1$ = 3.2, $pKa_2$ = 9.2, and $pKa_3$ = 12.3 at room temperature[28], while the allopurinol drug has only one dissociation constant pKa = 9.4[29]. At neutral pH 7, the predominant guanine species is neutral guanine molecule (Supplementary Fig. 16a), and the species of neutral allopurinol molecule is dominant (Supplementary Fig. 17a). The supersaturation $\sigma$ of guanine or allopurinol growth solution was thus calculated based on the following relationship:

$$\sigma = \frac{\Delta\mu}{k_B T} = \ln\left(\frac{C_s}{C_e}\right) \quad (21)$$

where $C_s$ is the solution concentration of guanine or allopurinol, and $C_e$ is the corresponding equilibrium concentration.

**2.2 Cooperative model of step pinning and kink blocking.** *In situ* AFM measurements of step advancement on the basal $(20\bar{2})$ surface revealed a "dead zone" for layer growth at low supersaturation. In this regime of solute concentration, we observed stagnant layers (i.e. $v$ = 0 nm/s), the presence of corrugated steps, and protrusions on step edges. These observations are consistent with a step-pinning mechanism of growth inhibition.[30,31]

The step-pinning mode of action proposed in the Cabrera−Vermilyea (C-V) model[30] considers the adsorption of inhibitors on the terrace and the creation of an adsorbate-to-adsorbate gate ($\Delta x$), which impedes the advancement of steps by imposing step curvature, thus increasing the chemical potential of curved steps due to the Gibbs-Thomson effect. According to the Gibbs-Thomson relation, the step curvature of radius $R$ is in equilibrium with a solution of activity $a_{e,R}$, which is greater than the equilibrium activity of a straight step $a_e$ by

$$\ln\left(\frac{a_{e,R}}{a_e}\right) = \frac{\Omega\alpha}{Rk_B T} \quad (22)$$

where $\alpha$ is the surface free energy of the step edge per unit step height, $\Omega$ is the molecular volume in the crystal, $k_B$ is Boltzmann's constant, and $T$ is the absolute temperature. If the spacing between any two impurities, $\Delta x$, is less than $2R_c$ (where $R_c$ is the critical radius), the motion of the step is thermodynamically prevented (Fig. 4a). Because of the inverse proportionality of $R_c$ against $\sigma$, an increase of growth solution supersaturation reduces $R_c$; and when the supersaturation increases above a critical value (i.e. $\sigma_d$ , the width of dead zone), the condition $\Delta x > 2R_c$ enables step segments to penetrate the inhibitor fence and growth recovers.

The average spacing distance between inhibitors, $\Delta x$, is related to their surface coverage $\theta_i$ by the relationship[32,33]

$$\Delta x \propto \left(\frac{1}{n_i}\right)^{0.5} = \frac{1}{\sqrt{\theta_i / S_0}} \quad (23)$$

where $n_i$ is the surface concentration of adsorbed inhibitors and $S_0$ is the area per adsorption site ($S_0 \cong 0.42$ nm$^2$). Assuming a Langmuir adsorption relationship between $\theta_i$ and the concentration of inhibitors in solution, $C_i$,[32,33], the surface coverage can be described by

$$\theta_i = \frac{K_i C_i}{1 + K_i C_i} \quad (24)$$

where $K_i$ is the Langmuir adsorption constant. Combining Equations 22-24, the critical supersaturation, $\sigma_d$, for the "dead zone" of growth is given by

$$\sigma_d = \ln(\frac{a_{e,R}}{a_e}) = \frac{2\Omega\alpha}{\mathrm{B}k_B T (S_0)^{0.5}} \sqrt{\frac{K_i C_i}{1+K_i C_i}} \quad (25)$$

where B is the proportionality constant reflecting the effects of sticking probability, geometric factors, and percolation threshold.[33] This expression for $\sigma_d$ is identical to the one derived by Weaver and De Yoreo.[32,33] From Equation 25, the solution activity of a curved step at equilibrium with radius $R$ is

$$a_{e,R} = a_e \exp\left( \frac{2\Omega\alpha}{Bk_B T (S_0)^{0.5}} \sqrt{\frac{K_i C_i}{1+K_i C_i}} \right) \quad (26)$$

At a solution activity $a > a_{e,R}$, any step with radius of curvature $R$ grows with velocity

$$v_R = \beta\Omega\left[ (a_{(u^-)} a_{(NH_4^+)})^{0.5} - (a_{e,R(u^-)} a_{e,R(NH_4^+)})^{0.5} \right] \quad (27)$$

where β is the kinetic coefficient.[34,35] At neutral conditions (pH 7), the activity of urate is equal to ammonium, thus the step velocity can be re-written as

$$v_R = \beta\Omega\left[ a_{(u^-)} - a_{e(u^-)} \exp\left(2\Omega\alpha / [\mathrm{B}k_B T (S_0)^{0.5}][K_i C_i / (1+K_i C_i)]^{0.5}\right) \right] = v_0 \frac{\beta}{\beta_0} \frac{a_{(u^-)} - a_{e(u^-)} \exp\left\{2\Omega\alpha / [\mathrm{B}k_B T (S_0)^{0.5}][K_i C_i / (1+K_i C_i)]^{0.5}\right\}}{a_{(u^-)} - a_{e(u^-)}} \quad (28)$$

where $v_0$ is the step velocity of straight step given by

$$v_0 = \beta_0 \Omega \left[ a_{(u^-)} - a_{e(u^-)} \right] \quad (29)$$

Combining equations 28 and 29 and assuming no change in β between $v_R$ and $v_0$, i.e. $\beta = \beta_0$, we obtain the following relationship

$$\frac{v_R}{v_0} = \frac{a_{(u^-)} - a_{e(u^-)} \exp\left\{2\Omega\alpha / [\mathrm{B}k_B T (S_0)^{0.5}][K_i C_i / (1+K_i C_i)]^{0.5}\right\}}{a_{(u^-)} - a_{e(u^-)}} \quad (30)$$

According to equation 30, for a given supersaturation the velocity $v_R$ of a step with curvature $R$ imposed by step pinners decreases monotonically, ranging from $v_0$ at $C_i = 0$ and $R = \infty$ to zero with increasing $C_i$.

The concentration of inhibitor (minor tautomer) increases linearly with solution concentration. The derived step velocity based on equation 30 shows no decline as the suepersatuation of growth solution increases, contradicting our observed change in step velocity (Figs. 2c and 4c). This suggests that the tautomer inhibition of step advancement involves another mode of action beyond step pinning. To this end, we posit a second common mechanism, kink blocking, which reduces the kinetic coefficient, β. As illustrated in Fig. 4b, we postulate the inhibitor (minor tautomer) can be incorporated competitively with solute (major tautomer) into kink sites.

To consider the reduction of the kinetic coefficient, we assumed the inhibitor adsorption inactivates kink sites using expressions derived by Weaver *et al.*[33] and Ma *et al.*[36], which account for the reduced β by an inhibitor(s). The density of available kink sites, $n_k$, in the presence of inhibitor can be expressed by the following

$$n_k = n_{k,0} - \frac{a}{\Delta x} \quad (31)$$

where $n_{k,0}$ is the density of the available kink sites in the "pure" system and $a$ is the molecular spacing along the step edge. Note that $n_{k,0}$ is a hypothetical case wherein only urate solute is present, which is not possible at neutral pH. The kinetic coefficient β is proportional to $n_k$

$$\frac{\beta}{\beta_0} = \frac{n_k}{n_{k,0}} = 1 - \frac{a}{n_{k,0}\Delta x} \quad (32)$$

where $\beta_0$ is the kinetic coefficient in the absence of impurity. Taking $\Delta x = 2R_c$ and combining equations 22-24, the reduction of kinetic coefficient, $\beta/\beta_0$, can be expressed as a function of concentration of inhibitor, $C_i$

$$\frac{\beta}{\beta_0} = 1 - \frac{a}{n_{k,0}B(S_0)^{0.5}}\sqrt{\frac{K_iC_i}{1+K_iC_i}} \quad (33)$$

A general expression is derived by combining equations 28-29 and 33, which can account for the reduction of the kinetic coefficient due to the poisoning of kink sites and a reduction in the thermodynamic driving force due to step curvature imposed by the adsorbed inhibitor on terraces.

$$\frac{v_R}{v_0} = \frac{1}{a_{(u^-)} - a_{e(u^-)}}\left(1 - A_1\sqrt{\frac{K_iC_i}{1+K_iC_i}}\right)\left\{a_{(u^-)} - a_{e(u^-)}\exp\left(A_2\sqrt{\frac{K_iC_i}{1+K_iC_i}}\right)\right\} \quad (34)$$

$$A_1 = \frac{a}{n_{k,0}B(S_0)^{0.5}} \quad (35)$$

$$A_2 = \frac{2\Omega\alpha}{Bk_BT(S_0)^{0.5}} \quad (36)$$

**2.3 Correlation of step velocity with concentration of kink blocker.** According to the Bliznakov growth rate expression[37,38], the effect of adsorption of kink blocker (B) on retardation of step velocity, $v_B$, can be quantitatively described by[36,39]

$$v_B = v_0 - (v_0 - v_{max})\theta_B \approx v_0(1 - \xi\theta_B) \quad (37)$$

where $\theta_B$ is the fractional surface coverage of impurity, $v_{max}$ is the step velocity at maximum inhibitor coverage (i.e. $\theta_B \rightarrow 1$), and $\xi$ is a parameter ($0 < \xi < 1$) accounting for the fraction of kinks accessible to the adsorbing inhibitors. The latter is limited owing to spatial overlap between adsorbed inhibitor molecules, fast kink dynamics, and/or alternative mechanisms.[36,40] The parameter $\theta_B$ is often estimated by assuming a Langmuir adsorption isotherm with finite number of equivalent adsorption sites, no interactions between adsorbed molecules, a desorption rate independent of the solution concentration of the inhibitor, and equilibrium between inhibitors on the surface and in the bulk,[36]

$$\theta_B = \frac{K_BC_B}{1+K_BC_B} \quad (38)$$

where $K_B$ is the Langmuir adsorption constant of kink blocker B, and combining equations 37 and 38 we obtain

$$v_B = v_0\left(1 - \xi\frac{K_BC_B}{1+K_BC_B}\right) \quad (39)$$

Equation 39 illustrates only the coefficient of proportionality reduces by a factor dependent of $C_B$ and predicts $v_B$ decreases monotonically from $v_0$ to its saturation value $v_{max}$ with increasing $C_B$.

Equation 37 combined with Chernov's expression of elementary step velocity[39] results in the interpretation that the apparent step kinetics coefficient is modulated by the presence of impurities adsorbing into kinks:

$$v_B = \Omega(a-a_e)\beta_i = \Omega(a-a_e)\beta_0\Theta = v_0\Theta \quad (40)$$

where $\beta_i$ is the step kinetic coefficient in the presence of impurity i, and $\Theta$ (=1- $\xi\theta_B$) is the factor or modulator regulating the step velocity from $v_0$ ($\theta_B = 0$) to $v_{max}$ ($\theta_B = 1$).[39] When the impurity first adsorbs onto the surface and diffuses two-dimensionally across the terrace or ledge to enter into a kink, the modulator $\Theta$ becomes (=$\beta_i/\beta_0$=1-$a/n_{k,0}\Delta x$).[33,39] In this case, step velocity $v_B$ can be expressed by

$$v_B = v_0\left(1 - A_1\sqrt{\frac{K_iC_i}{1+K_iC_i}}\right) \quad (41)$$

Equation 41 is the simplified one from equation 36 when the reduction of kink density is attributed to inhibitors (i.e. kink blockers).

**2.4 Variation of the surface free energy of step edges.** The measured critical size of 2D islands (Supplementary Fig. 18c) displays continuously positive deviation from the Gibbs-Thomson relation before reaching the maximum of step velocity and subsequently rapid decline. The trend is much more evident when the calculated surface free energy, α, was plotted as a function of the concentration of urate (Supplementary Fig. 21). It shows the linear increase with urate concentration and then a sharp decrease. Given the presence of equimolar NaCl in the ammonium urate growth solution, the increase of crystallization driving force (i.e. solute concentration) concomitantly elevates the concentration of salts. It is well-known that the addition of most inorganic salts to water raises the surface tension approximately linearly with the salt concentration[41-44], assessed by Gibbs adsorption equation[45]. We postulated this initial increase of surface tension is due to the effect of salts.

The surface free energy of step edge decreases dramatically with a decline in step velocity and increased rate of 2D nucleation (Fig. 2c and Supplementary Fig. 18). These observations appear to suggest a decrease of surface free energy of step edges occupied partially by the minor tautomer, $[DKE\text{-}N_9]^-$. We assume that the density of kinks occupied by adsorbed inhibitors, $n_{k,i}$, is equal to the number of molecules adsorbed at the kinks. According to Gibbs equation of adsorption[45], we have

$$\frac{n_{k,i}}{hb}d\mu_i = -d\alpha \quad (42)$$

where h is the step height, b is the width of an adsorption site, and $\mu_i$ is the chemical potential of urate in solution. The kinks occupied by inhibitors are

$$n_{k,i} = n_{k,0} - n_k = \frac{a}{B(S_0)^{0.5}}\sqrt{\frac{K_iC_i}{1+K_iC_i}} \quad (43)$$

This expression can be rearranged as the following equation

$$\frac{n_{k,i}}{n_{k,0}} = 1-\frac{\beta}{\beta_0} = \frac{a}{n_{k,0}B(S_0)^{0.5}}\sqrt{\frac{K_iC_i}{1+K_iC_i}} = A_1\sqrt{\frac{K_iC_i}{1+K_iC_i}} \quad (44)$$

Equation 44 reflects the fraction of kink sites occupied by the inhibitors relative to the hypothetical density of kink sites, $n_{k,0}$, in pure solution.

The chemical potential of urate can be written as

$$\mu_i = \mu_i^0 + k_BT\ln C_i \quad (45)$$

where $\mu_i^0$ is the standard value of $\mu_i$. Combining equations 42-45, the change of surface free energy of a step edge can be expressed by the following:

$$-\Delta\alpha = \int_0^{\mu_i}\frac{a}{Bhb(S_0)^{0.5}}\sqrt{\frac{K_iC_i}{1+K_iC_i}}d\mu_i = \frac{ak_BT}{Bhb(S_0)^{0.5}}\int_0^{C_i}\sqrt{\frac{K_i}{(1+K_iC_i)C_i}}dC_i \quad (46)$$

and

$$-\Delta\alpha = \frac{ak_BT}{\mathrm{Bhb}(\mathrm{S}_0)^{0.5}}\int_0^{C_\mathrm{i}} \frac{1}{\sqrt{(1+K_\mathrm{i}C_\mathrm{i})K_\mathrm{i}C_\mathrm{i}}} d(K_\mathrm{i}C_\mathrm{i}) = \frac{2ak_BT}{\mathrm{Bhb}(\mathrm{S}_0)^{0.5}} \mathrm{asinh}(\sqrt{K_\mathrm{i}C_\mathrm{i}}) \quad (47)$$

On $(20\bar{2})$ surfaces of $NH_4HU$ crystals with a step edge along the <101> direction, the molecular spacing, $a$, along the step edge is around 0.56 nm with a width b ≅ 0.35 nm in the [010] direction (Fig. 4i), and the step height h is 0.9 nm (Supplementary Fig. 12b). The proportional coefficient B has an order of magnitude of 1.[32,33] The inhibitor concentration spans from 0.8 to 1.5 mM, and the adsorption constant $K_\mathrm{i}$, fitted from normalized $v/v_0$ plot (Fig. 4c), is around 1.9 $mM^{-1}$. With these values of each parameter, we obtain that dimensionless coefficient accounting for the adsorption of inhibitors at kinks, asinh $(\sqrt{K_\mathrm{i}C_\mathrm{i}})$, is in the range of 1.2 to 1.7. The estimated decrease in step edge surface free energy is ca. 23 $J/nm^2$, consistent with the measured value of 20-30 $J/nm^2$ (Supplementary Fig. 18c).

The combination of equations 34-36 and 47 leads to

$$\frac{v_R}{v_0} = \frac{1}{a_{(u^-)} - a_{e(u^-)}}\left(1 - \mathrm{A}_1\sqrt{\frac{K_\mathrm{i}C_\mathrm{i}}{1+K_\mathrm{i}C_\mathrm{i}}}\right)\left\{a_{(u^-)} - a_{e(u^-)}\exp\left(\mathrm{A}_2^{'}\left[1-\mathrm{A}_3\mathrm{asinh}(\sqrt{K_\mathrm{i}C_\mathrm{i}})\right]\sqrt{\frac{K_\mathrm{i}C_\mathrm{i}}{1+K_\mathrm{i}C_\mathrm{i}}}\right)\right\} \quad (48)$$

and

$$\mathrm{A}_2^{'} = \frac{2\Omega\alpha_0}{\mathrm{B}k_BT(\mathrm{S}_0)^{0.5}} \quad (49)$$

$$\mathrm{A}_3 = \frac{2ak_BT}{\mathrm{Bhb}\alpha_0(\mathrm{S}_0)^{0.5}} = \mathrm{A}_2^{'}\frac{a(k_BT)^2}{\Omega\mathrm{hb}(\alpha_0)^2} \quad (50)$$

where $\alpha_0$ is the surface free energy of step edge in "pure" solution. Equation 46 accounts for step curvature, kink sites poisoning, and decrease of surface free energy by the native growth inhibitor of urate (i.e. minor tautomer $[DKE\text{-}N_9]^-$).

**2.5 Propagation of steps with low kink density and kink-limited growth model.** The experimentally measured step velocity (Fig. 2c) was normalized by the approximate $v_0$ with the kinetic coefficient $\beta_0$ in pure solution estimated at high pH and plotted as a function of supersaturation, σ. In situ AFM measurements of $NH_4HU$ $(20\bar{2})$ surface growth reveal serrated step morphology, and the velocity of step advancement exhibits a zero intercross at solubility. These observations suggest a step-pinning mode of action. According to the characteristic increasing trend of step velocity against solution supersaturation for typical step-pinning inhibition,[46] we estimated the kinetic coefficient of urate in the absence of impurity to be 1.4 to 2.8 (Fig. 2h). The normalized $v/v_0$ data cannot be fitted by equation 30 which does not consider the decrease of kink sites but can be fit using equation 34 accounting for the reduction of kinetic coefficient β and kink sites by tautomer inhibitor, which gives the fitting values of $A_1$, $K_\mathrm{i}$ and $A_2$ (Supplementary Table 5). Because solution pH may affect the accuracy of kinetic coefficient estimation for urate in the absence of impurity (i.e. $\beta_0$), we performed sensitivity analyses in a number of tested $\beta_0$ values to examine its influence on the fitting results of $A_1$, $K_\mathrm{i}$ and $A_2$. The results are shown in Supplementary Fig. 18d and Supplementary Table 5. Although the values of $\beta_0$ affects absolute values of $1-\beta/\beta_0$, the general trend remains, i.e. the kink sites occupied by inhibitors increases rapidly with solution supersaturation or concentration of minor tautomer inhibitor.

When the surface coverage of inhibitors, i.e. $1-\beta/\beta_0$, reaches a level of around 90%, the normalized step velocity $v/v_0$ drops sharply with increasing supersaturation. This remarkable decline may be due to the large fraction of kink sites occupied by the tautomers leading to crystal growth at low kink density (surface growth controlled by kink-limited growth[47]) or the increase of thermodynamic stability of step edges by incorporation of minor tautomer into the lattice. We observed a decline, instead of increase, of the critical size and thus decrease of surface free energy according to the Gibbs-Thomson effect, and no direct evidence supports the increasing chemical potential of step edges by inhibitor incorporation; thus we posit the

fraction of kink sites occupied by minor tautomer alters the density of available kink sites. As shown in Supplementary Fig. 18e, the trend of declining $v/v_0$ over supersaturation can be reasonably fit by a kink-limited growth model (i.e., $v/v_0 = 1+b_1C_i-b_2C_i^2$), with values of $b_1 = -1.0$ and $b_2 = -0.24$. The kink-limited growth model was initially proposed by De Yoreo *et al.*[47] in the explanation of unexpected inhibition behavior of $Sr^{2+}$ on calcite step growth where the step speed drops precipitously into a dead zone when the concentration of $Sr^{2+}$ is above a threshold. Because of low kink density, the propagation of steps is no longer controlled by the solute attachment to available kinks but controlled by kink formation.[47,48] The kink-limited growth mode also predicts the cessation of step advancement occurring at the nearly linear relationship between concentrations of inhibitor and solute. This is consistent with the ammonium urate growth medium wherein the concentration of minor tautomer is linearly proportional to urate concentration. Indeed, we observed nearly complete growth cessation at 9.0 mM urate.

**3. Supplementary Figures**

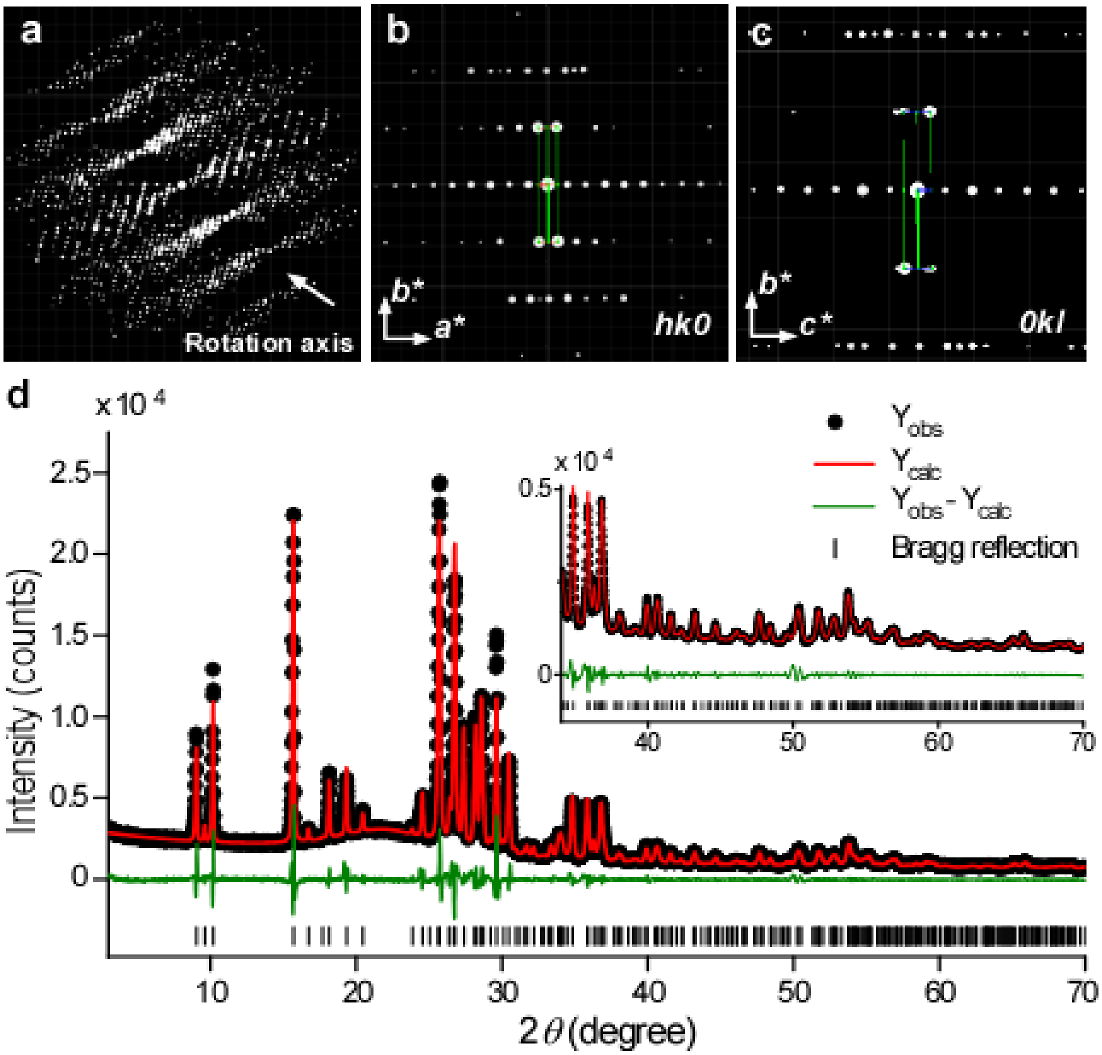


**Supplementary Fig. 1. Structure determination of $NH_4HU$ crystals.** (**a**) 3D reciprocal lattice of $NH_4HU$ reconstructed from 3D ED data. (**b** and **c**) Two-dimensional slices from the 3D reciprocal lattice showing the (hk0) and (0kl) reciprocal planes. The following reflection conditions can be deduced: $hkl$: $h+k = 2n$; $hk0$: $h+k = 2n$; $h0l$: $h = 2n, l = 2n$; $0kl$: $k = 2n$. (**d**) Rietveld refinement profile from capillary PXRD collected using a variable counting time strategy with experimental intensities (black dots), calculated intensities (red line), and a difference plot (green line). The tick marks represent the Bragg reflections. Inset: enlarged region in the range 35° < 2θ < 70°.

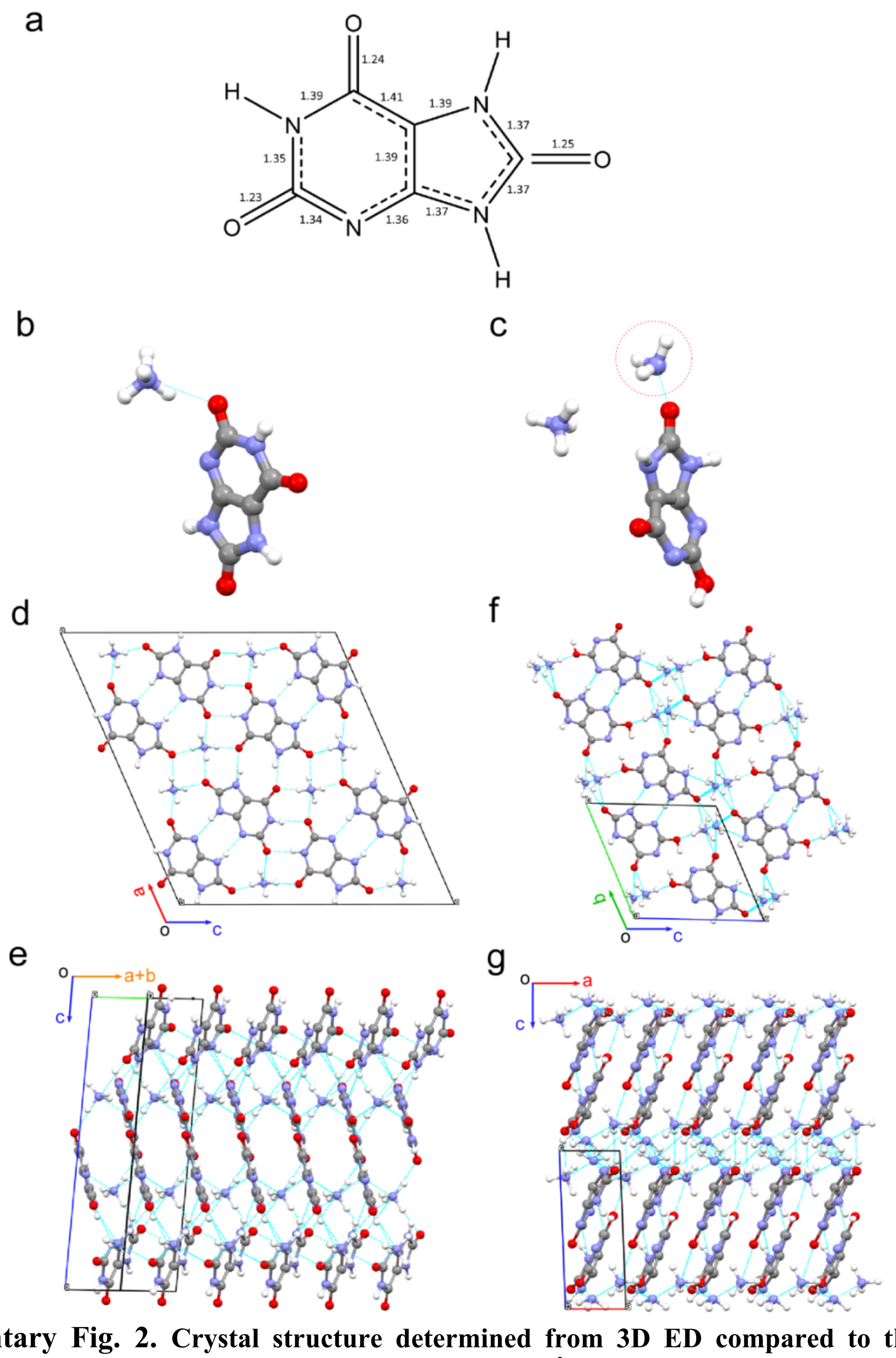


**Supplementary Fig. 2. Crystal structure determined from 3D ED compared to the proposed structure in literature.** (**a**) Geometric bond length (in Å) of urate within the $NH_4HU$ crystal structure obtained from 3D ED. In the structure, the resonant effect of the rings reduces the bond length difference between C-C and C-N bonds. Notably, the C-N bond length of the N atom without H attached (1.33(1) Å and 1.35(1) Å) is shorter than others (1.35(1) Å-1.40(1)Å), which confirms the hydrogen positions because this C-N bond is closer to C-N double bond. (**b** and **c**) Comparison of the molecular geometry of one asymmetric unit in the structure obtained from 3DED (b) and from PXRD in literature (c)[49]. The stoichiometric ratio of urate and ammonium was determined to be 1:1 in our solved crystal structure, consistent with elemental analysis in

Supplementary Table 2. (c) The dashed circle highlights an additional $NH_3$ atom in the unit cell proposed by Friedel et al.[49] that does not align with elemental analysis. (**d** and **e**) Molecular packing of the crystal structure determined in this study viewed down the (d) [010] and (e) [$1\bar{1}0$] directions. In the a-c plane the urate molecules have hydrogen bonding interactions with both neighboring urate molecules and the ammonium ions. The urate molecules are stacked along b-axis via π-π stacking. (**f** and **g**) Molecular packing of crystal structure proposed by Friedel et al. (P-1 space group) when viewed down the (f) [100] and (g) [010] directions. The molecular conformation of urate in the previously reported structure is slightly bent, i.e. atoms in the five- and six-membered rings are not in the same plane.

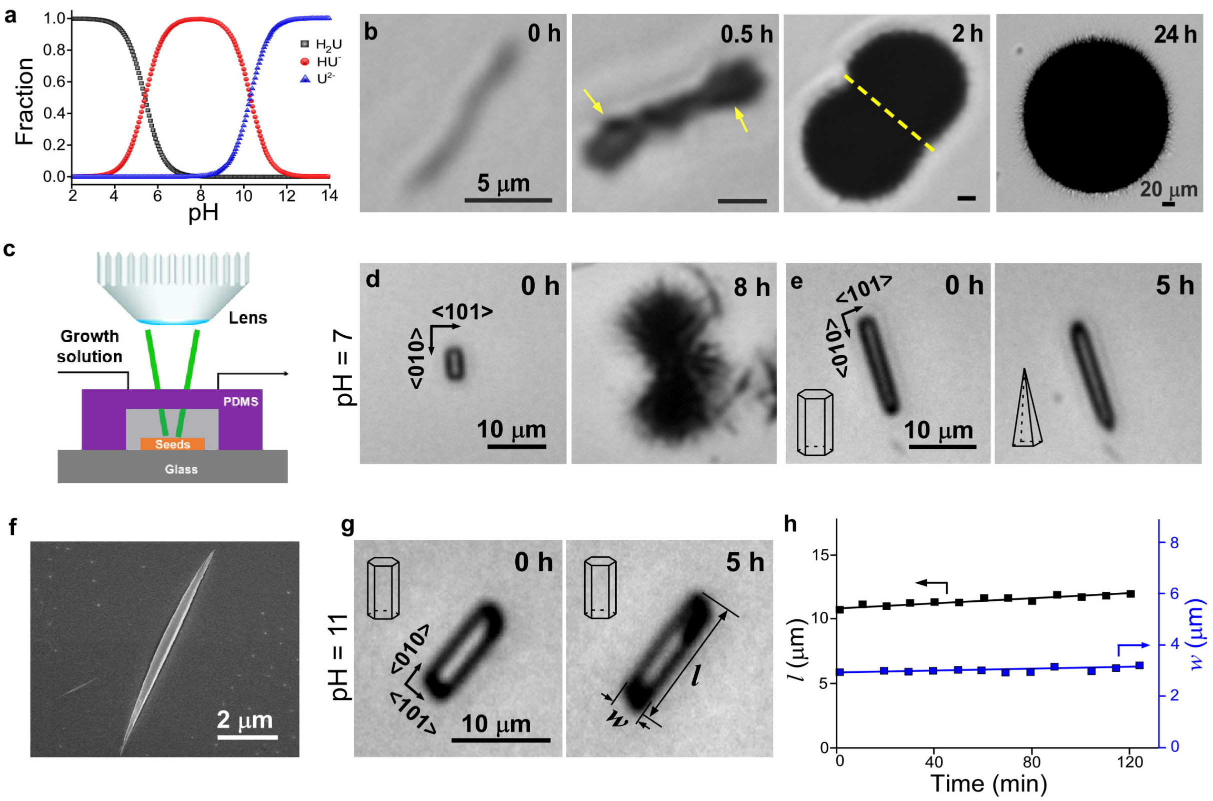


**Supplementary Fig. 3. Effect of pH on $NH_4HU$ crystal growth and morphology.** (**a**) Solution speciation of urate ($pKa_1$=5.4 and $pKa_2$=10.3) as a function of solution pH: uric acid ($H_2U$, gray), monovalent urate anion ($HU^-$, red), and divalent urate anion ($U^{2-}$, blue).[26] (**b**) Time-resolved optical micrographs of $NH_4HU$ dendritic growth showing intermediate dumbbell crystals that evolve into the final spherulite-like crystals. These experiments were performed using sodium urate and ammonium chloride as reagents. (**c**) Schematic of a microfluidic device where seed crystals (orange) are embedded into a poly(dimethylsiloxane) channel (purple). (**d**) Development of a dumbbell-shape crystal from a rod-like seed crystal in a growth solution at pH 7 and equimolar solute concentration (10 mM urate). (**e**) Time-resolved imaging at lower supersaturation (6 mM urate) showing the emergence of tapering in the $NH_4HU$ crystal seed due to the presence of $[DKE\text{-}N_9]^-$ inhibitor. (**f**) SEM image of a tapered crystal after growth in pH 7 solution (6 mM urate) for 10 h. (**g**) Microfluidics growth of a $NH_4HU$ seed crystal at pH 11 and 7 mM urate showing the absence of bending, branching, or tapering (*l* and *w* denote length and width, respectively). (**h**) Representative time-elapsed changes in crystal length (black) and width (blue) during *in situ* microfluidics

growth for images collected in panel g. The slope of these curves corresponds to the rate of growth reported in Figure 1g.

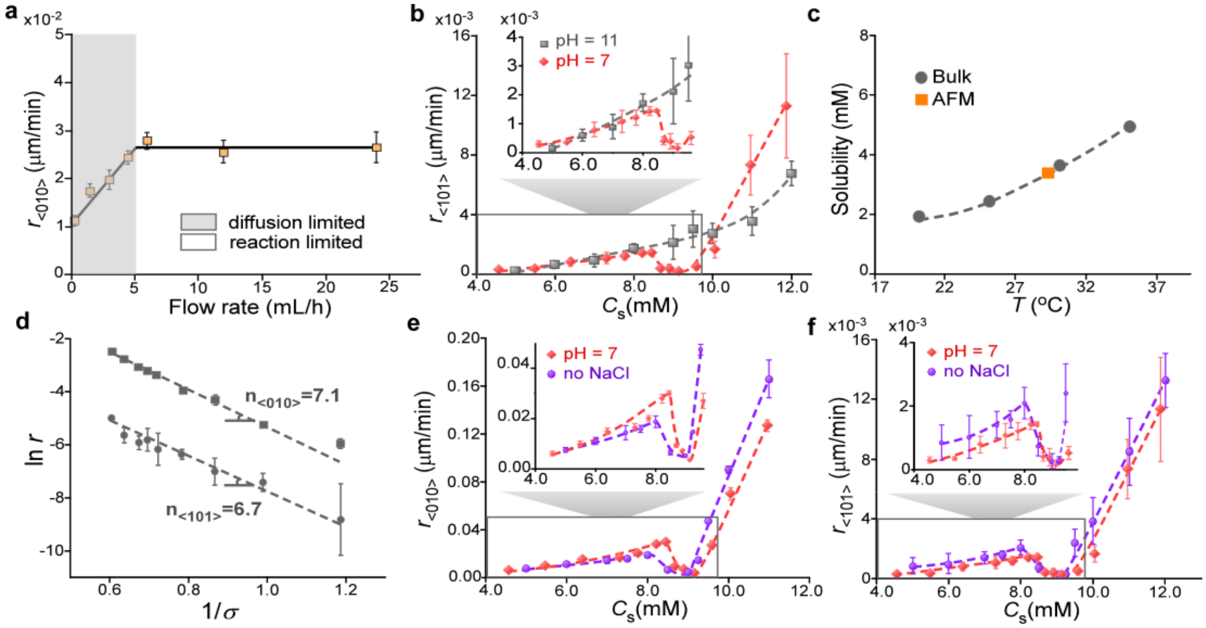


**Supplementary Fig. 4. Growth kinetics of $NH_4HU$ crystals in a microfluidic device.** (**a**) Effect of flow rate on $NH_4HU$ seeded growth in a solution with pH 7 and 8.2 mM urate (σ =1.3 at 21°C). Symbols are the average of 10 to 15 measurements for a single run and error bars span two standard deviations. The solid lines are interpolations to guide the eye. (**b**) The rate of growth along the <101> direction, $r_{<101>}$, at pH 11 (grey) and pH 7 (red) as a function of urate concentration. The trends are identical to $r_{<010>}$ reported in Fig.1g. Inset: enlarged region near growth cessation. Symbols are the average of 10 to 15 measurements for a single run and error bars span two standard deviations. Dash lines are interpolated to guide the eye. (**c**) The solubility of $NH_4HU$ crystals at pH 11 determined from bulk experiments and AFM step velocity measurements (Fig. 2h, inset). Data are the average of three independent measurements and error bars span two standard deviations (those not visible are smaller than the symbol size). (**d**) Linearized plot of growth rates $r_{<101>}$ and $r_{<010>}$ at pH 11 versus 1/σ reveals an exponential dependence on supersaturation ($r \propto \exp(-B/\sigma)$) where B is the thermodynamic parameter associated with the energy barrier to create a critical radius of 2D islands for the birth and spreading growth mechanism.[50,51] (**e** and **f**) Comparison of (e) $r_{<010>}$ and (f) $r_{<101>}$ at pH 7 in the absence of NaCl (purple) using uric acid and ammonium hydroxide as reagents, and in the presence of NaCl (red) using sodium urate and ammonium chloride as reagents. These experiments were conducted using different sources (uric acid and ammonium hydroxide) compared to conditions reported in the manuscript (using sodium urate and ammonium chloride) to show that the presence of NaCl (i.e. impurity) does not impact the trend in $NH_4HU$ growth kinetics.

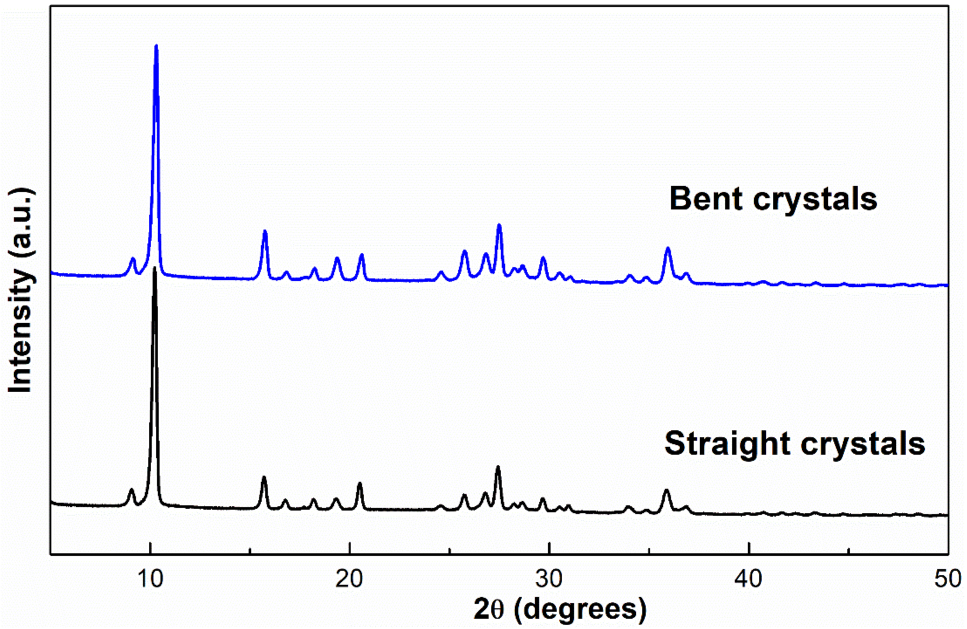


**Supplementary Fig. 5. Crystallinity of bent crystals.** Powder X-ray diffraction patterns of $NH_4HU$ bent crystals prepared at pH 7 (6-7 mM urate) versus straight crystals prepared at pH 11 (6 mM urate). There is no observable difference between straight and bend crystals, suggesting that bent crystals have identical phase composition compared to straight crystals.

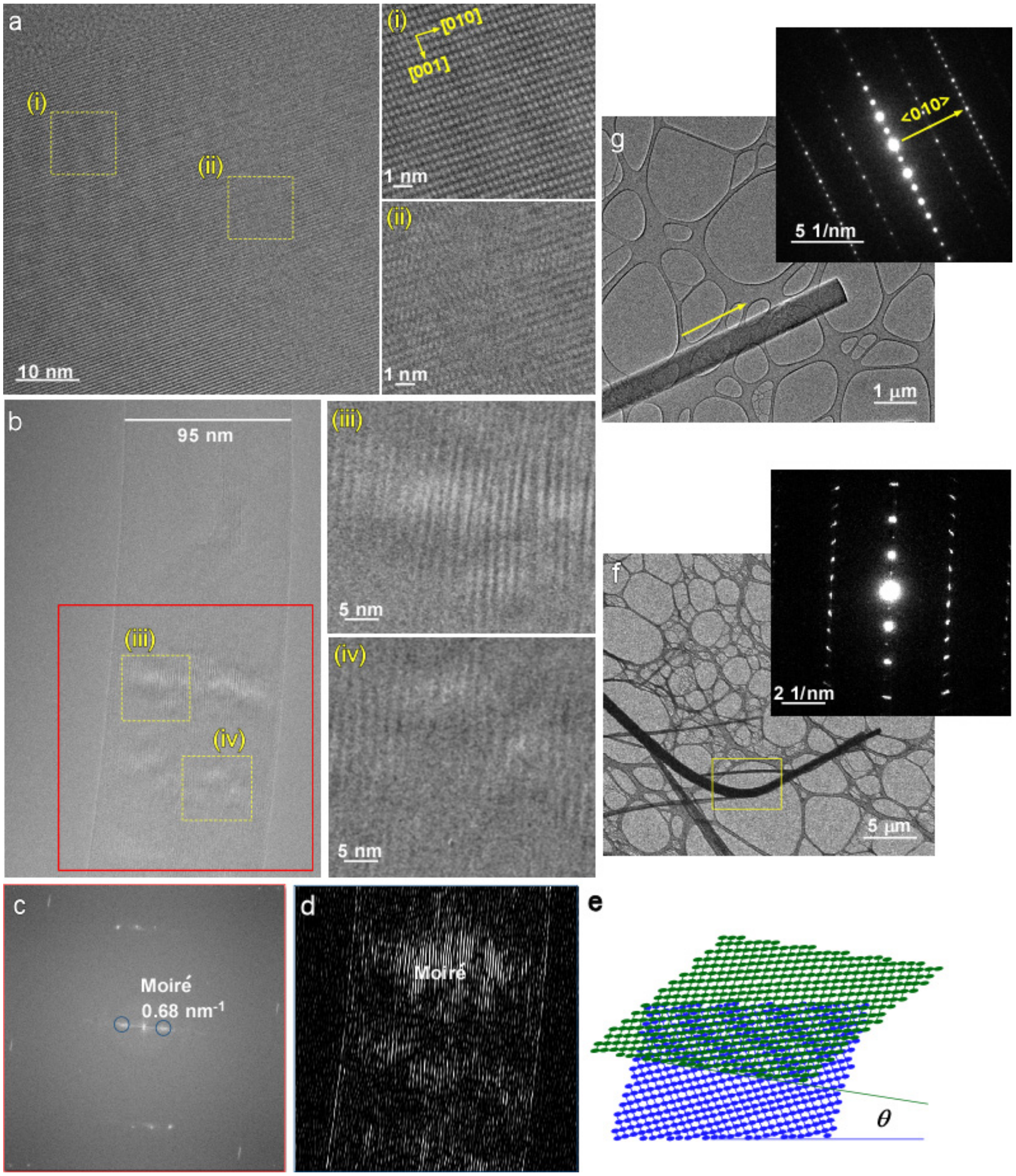


**Supplementary Fig. 6. Collection and analysis of high-resolution TEM images.** (**a**) Final high resolution image after overlapping 10 low dose images with a low fractionation technique in the TEAM 1 electron microscope with a Cc Corrector and a K2 Camera. This is achieved by using Nelsonian illumination producing a remarkably coherent ($\Delta E \leq 100$ meV), pencil-like illumination rod of a diameter that can be matched to the field of view (FoV) of the K2 camera ($10^6$ -$10^7$ Å$^2$), while detecting single electron scattering events and maintaining atomic resolution capability with a magnification-dependent image pixel size (ca. 0.3*0.3 Å$^2$). Low-dose experiments benefit from the ability to quickly change the beam current (< 1 s) by orders of magnitude without introduction of uncontrolled lens aberrations. It was observed that more than 10 dose fractionation images generate permanent damage to the actual sample. For this reason, the study was limited to 10 images meeting the critical accumulation dose of 4 $e^-$•Å$^{-2}$. Images *i* and *ii* are magnified areas. The crystal structure was solved from 3D ED data (Supplementary Table 1). All non-hydrogen atoms could be directly located from the structure solution. The hydrogen atoms were identified from the residual (Q)-

peaks in the difference Fourier maps during the structure refinements and subsequently refined without adding any restraints. There are one urate and one ammonium ions in the asymmetric unit (Supplementary Fig. 2). (**b**) Bent crystal after a dose fractionation technique (10 images). Images *iii* and *iv* are magnified areas showing atomic cluster arrangements and linear defects (edge dislocations). (**c**) Fourier transform of panel b showing reflections produced by Moiré fringes. (**d**) Inverse Fourier Transform image showing Moiré fringes. (**e**) Schematic of Moiré fringes**.** (**f** and **g**) TEM analysis of $NH_4HU$ crystals prepared at (f) pH 7 and (g) pH 11 where SAED patterns (insets) show the absence and presence of line broadening, respectively.

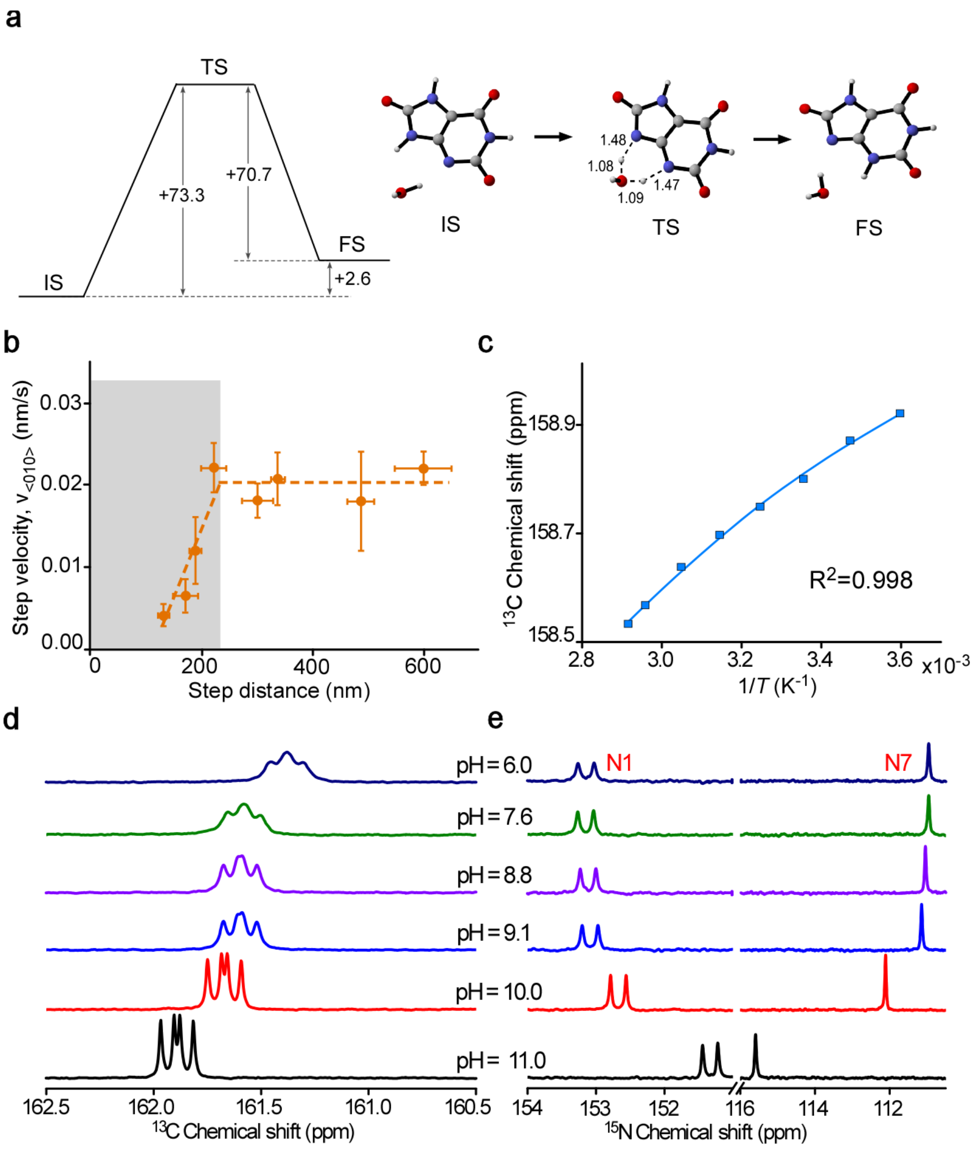


**Supplementary Fig. 7. Analysis of urate tautomer exchange dynamics.** (**a**) Idealized reaction pathway (left) showing the interconversion between $[DKE\text{-}N_3]^-$ (initial state, IS) and $[DKE\text{-}N_9]^-$ (final state, FS) via a transition state (TS) facilitated by water (Supplementary Movie 6). The reaction diagram is in Gibbs free energy (in kJ/mol) calculated using M06-2X[52,53] level of theory and the 6-311+G(d,p) basis set at 25 ºC and the respective fully optimized structures are shown on right with lengths of bonds that are involved in the transition state (in Å). (**b**) The dependence of step velocity $v_{<010>}$ from *in situ* AFM on interstep distance *l* at pH 7 and 5 mM urate is characteristic of step advancement as a result of solute incorporation via surface

diffusion rather than direct incorporation from solution to kink sites.[54-56] (**c**) Analysis of solution $^{13}$C NMR reveals a temperature-dependent trend in the carbonyl $^{13}$C chemical shift in 10 mM urate (2-$^{13}$C,1,3,7-$^{15}$N3 atomic labeled) in 95% (v/v) $D_2O/H_2O$ at pH 7. The solid line is the best fit to $\delta = \delta_{[\mathrm{DKE-N_3}]^-} + K(\delta_{[\mathrm{DKE-N_9}]^-} - \delta_{[\mathrm{DKE-N_3}]^-})/(1+K)$, where $\delta$ is chemical shift of carbonyl $^{13}$C resonance and $K$ is tautomeric equilibrium constant (Fig. 3f). (**d**) $^{13}$C-NMR spectra obtained from pH titration experiments in a 10 mM labeled urate-2-$^{13}$C,1,3,7-$^{15}N_3$ solution with pH adjustments by the addition of either 1.0 M $HCl_{(aq)}$ or 1.0 M $NaOH_{(aq)}$, showing gradual peak broadening with decreased alkalinity. (**e**) $^{15}$N-NMR spectra obtained from the same pH titration experiments showing the absence of N3 peak in the 6-membered ring of urate indicating a fast exchange of hydrogen atoms with $H_2O$.

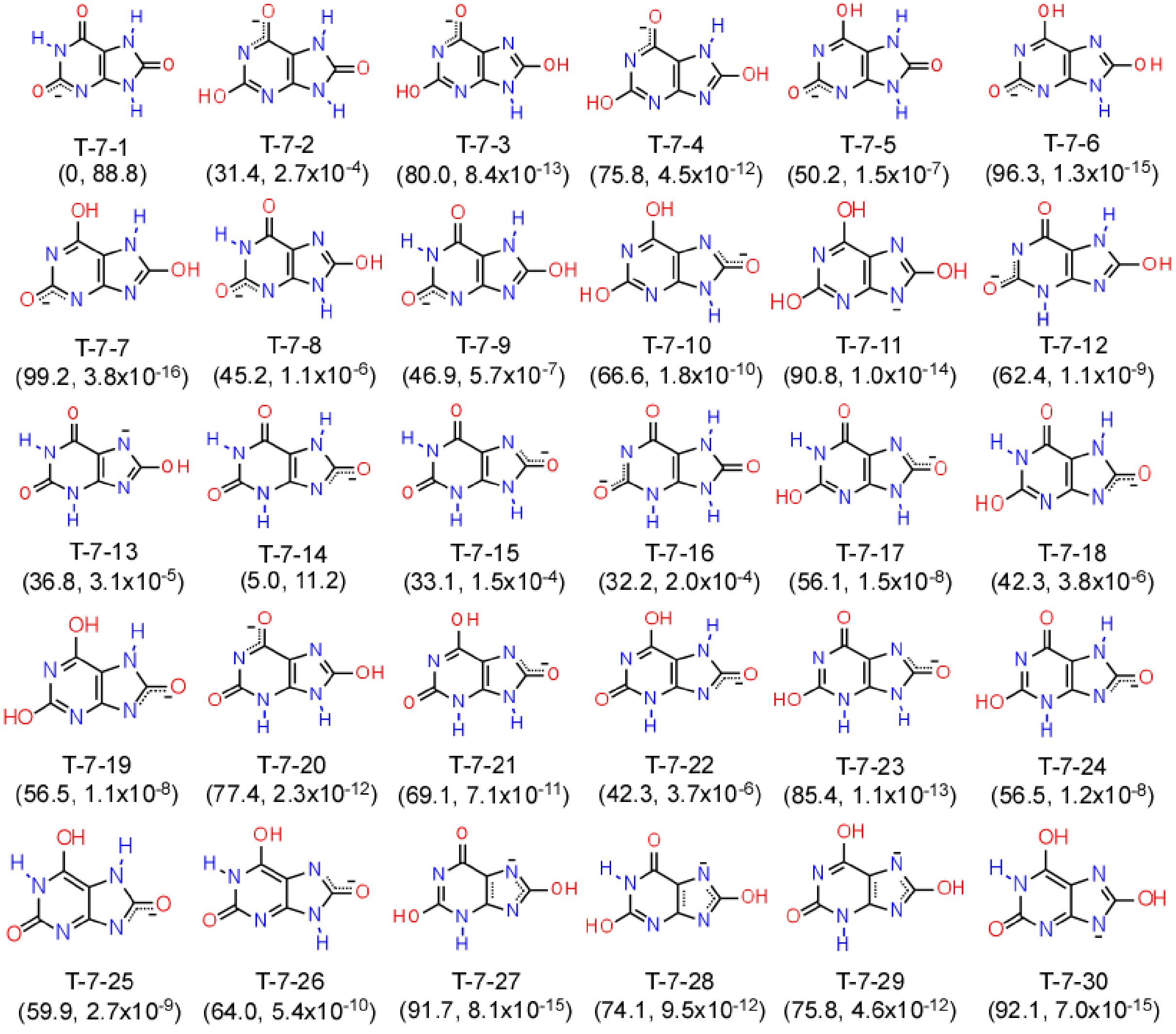


**Supplementary Fig. 8. First principles calculated structures, Gibbs free energy, and population of urate monovalent tautomer species at pH 7 and 25 °C.** Calculations were performed using CBS-QB3[57] level of theory. The term "T-7-x" represents a urate tautomer in aqueous solution at pH 7 labeled *x* starting from 1 to 30. The values in parentheses represent relative Gibbs free energy to the most stable tautomer (T-7-1) (kJ/mol) and Boltzmann population (%), respectively. The major tautomer $[DKE\text{-}N_3]^-$ corresponding to solute incorporated in the $NH_4HU$ crystals is T-7-1 and the minor tautomer $[DKE\text{-}N_9]^-$ corresponding to the growth inhibitor is T-7-14.

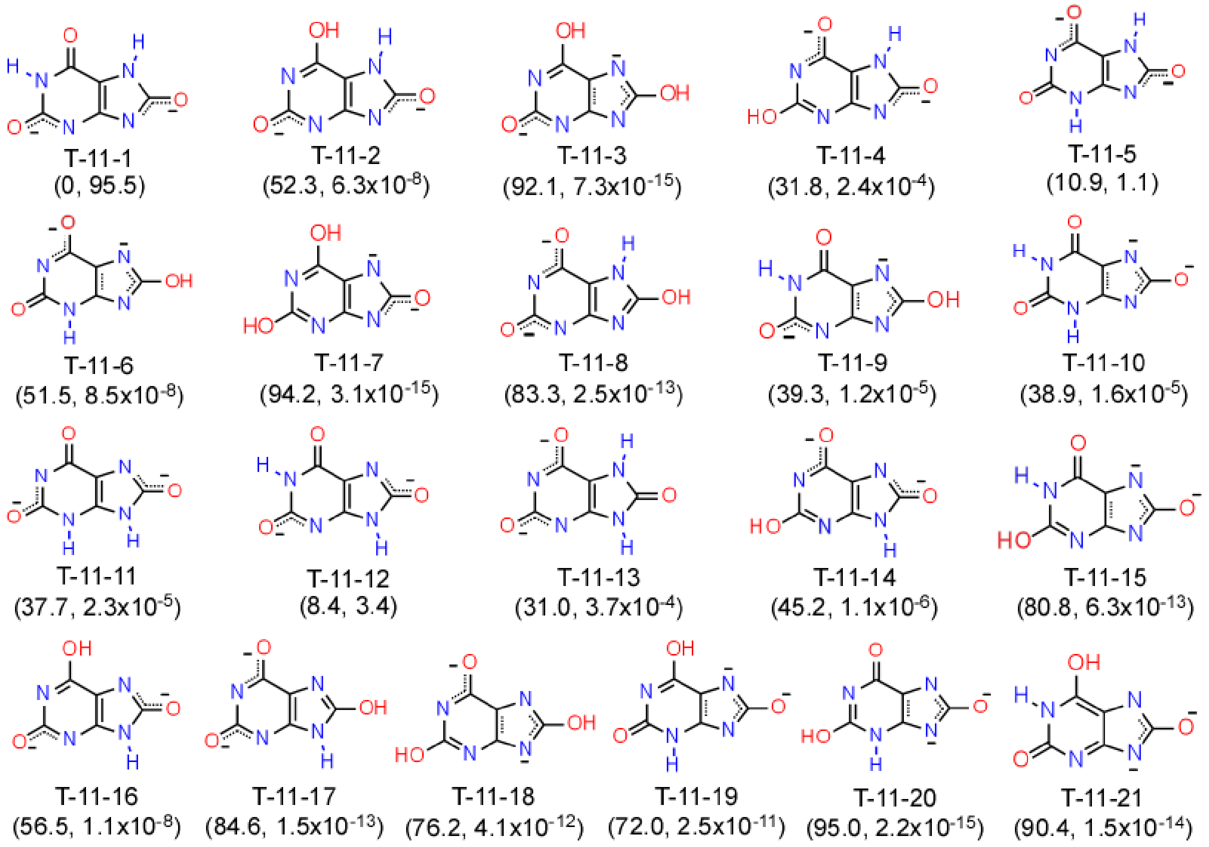


**Supplementary Fig. 9. First principles calculated structures, Gibbs free energy, and population of urate divalent tautomer species at pH 11 and 25 ºC.** The calculations were performed using CBS-QB3[57] level of theory. The term "T-11-x" represents a urate tautomer in aqueous solution at pH 11 labeled *x* starting from 1 to 21. The values in parentheses represent relative Gibbs free energy to the most stable tautomer (T-11-1) (kJ/mol) and Boltzmann population (%), respectively.

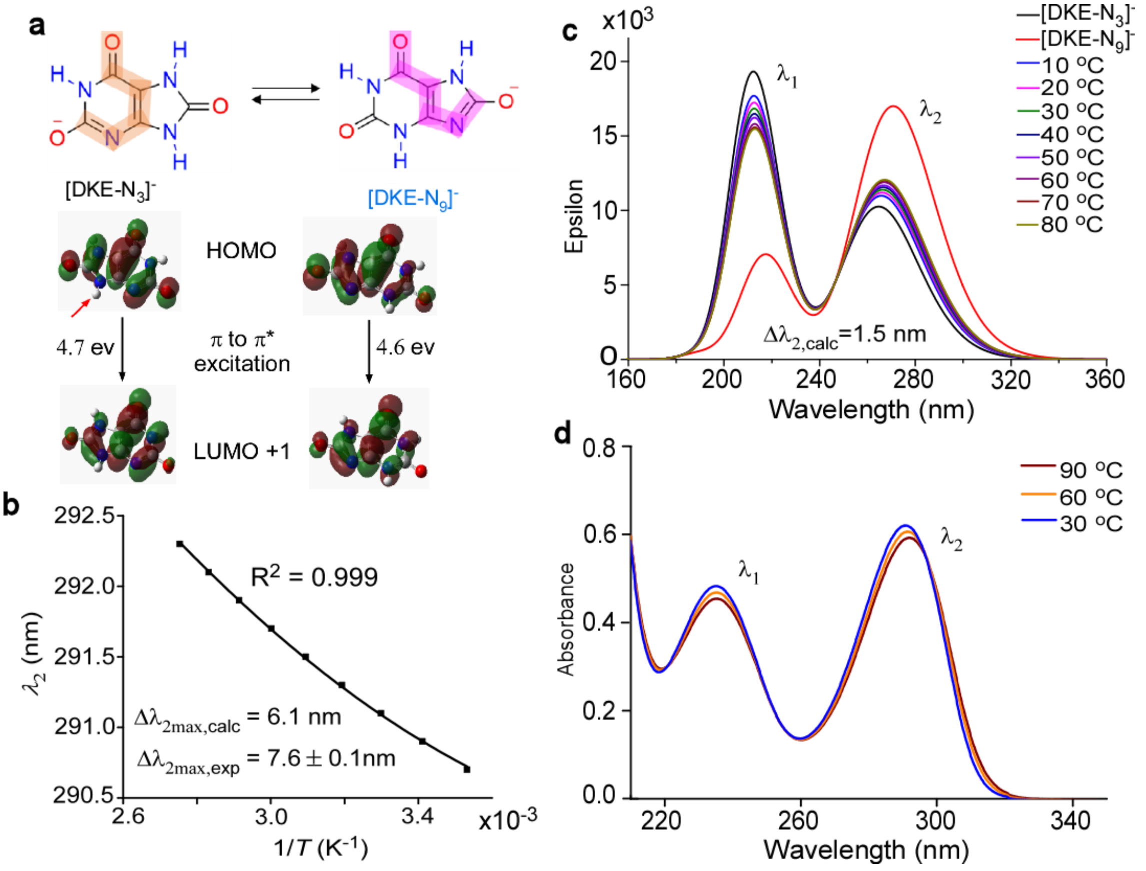


**Supplementary Fig. 10. Variable temperature UV-Vis spectra of urate solutions at pH 7.** (**a**) Origin of UV-Vis bands of diketo-enol tautomers. The computed highest occupied molecular orbital (HOMO) shows that the main difference between the $[DKE\text{-}N_3]^-$ and $[DKE\text{-}N_9]^-$ tautomers is due to the overlap of p-orbitals in the five member ring, while the lowest unoccupied molecular orbital (LUMO) of two tautomers remains similar. Differences in conjugated structure lead to a lower-energy UV–Vis absorption band (and higher wavenumber) for $[DKE\text{-}N_9]^-$. (**b**) Nonlinear dependence of the UV-Vis absorption peak $\lambda_2$ on temperature. The solid line is the best fit to $\lambda_2 = \lambda_{2,[DKE-N_3]^-} + K\Delta\lambda_{2,max}/(1+K)$ , where $\Delta\lambda_{2,max} = \lambda_{2,[DKE-N_9]^-} - \lambda_{2,[DKE-N_3]^-}$ and $K$ is the tautomeric equilibrium constant. The experimental UV-Vis wavelength difference between two tautomers $\Delta\lambda_{2max,exp} = 7.6 \pm 0.1$ nm is close to the DFT calculated value of 6.1 nm. (**c**) Results of DFT calculated (M06-2X/6-311+G(d,p) level) UV-Vis absorption spectra of $[DKE\text{-}N_3]^-$ (black line) and $[DKE\text{-}N_9]^-$ (red line) and simulated UV-Vis spectra of a combination of $[DKE\text{-}N_3]^-$ and $[DKE\text{-}N_9]^-$ at different temperatures. DFT predicts an identical bathochromic shift of the $\lambda_2$ band with a maximum shift, $\Delta\lambda_{2,calc}$ =1.5 nm, that is close to the experimental measurements (Fig. 3b). (**d**) The reversible shift of UV-Vis absorption $\lambda_2$ band upon decreasing temperature in an aqueous solution (pH 7, 1 mM urate) validates the equilibrium behavior of urate tautomers.

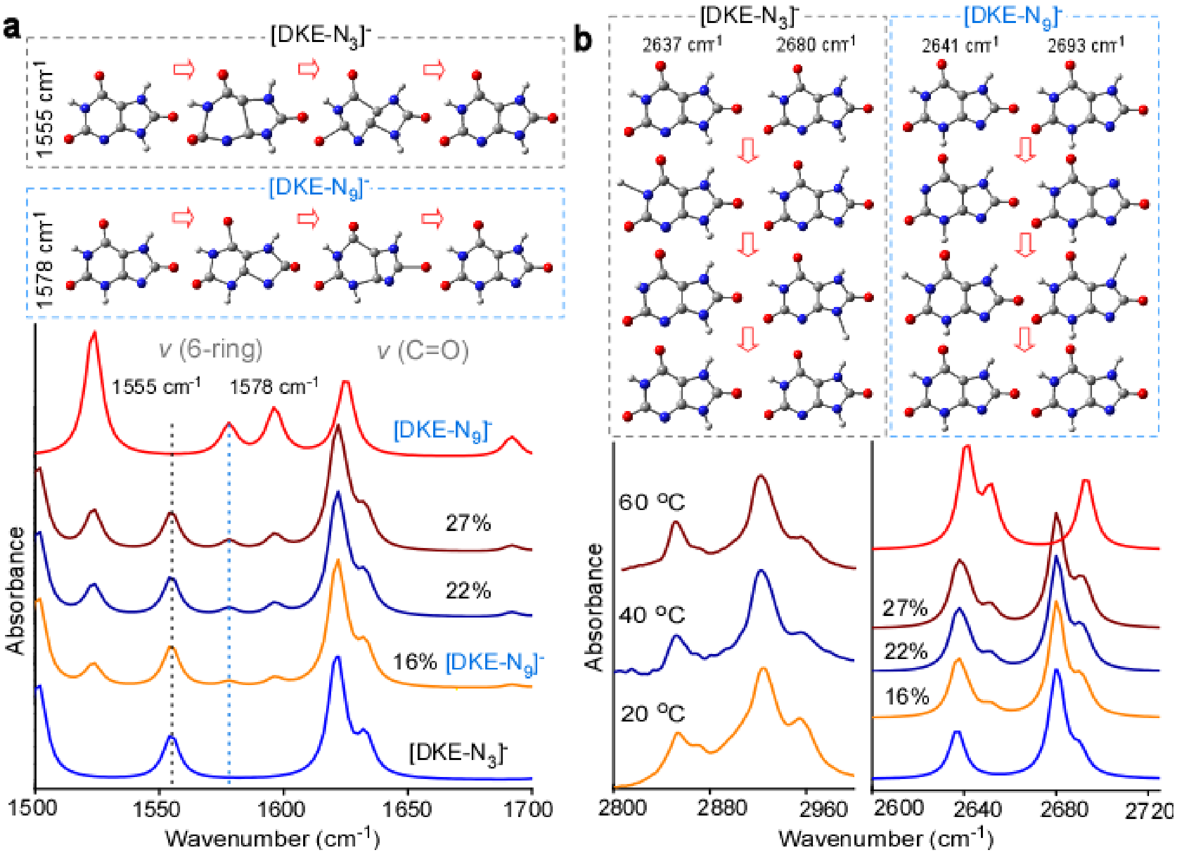


**Supplementary Fig. 11. Peak assignment of IR spectra at pH 7.** (**a**) (Top) Animation snapshots showing IR asymmetric stretching difference of the six-membered ring in $[DKE\text{-}N_3]^-$ (1555 $cm^{-1}$) and $[DKE\text{-}N_9]^-$ (1578 $cm^{-1}$) tautomers. (Bottom) DFT calculated IR spectra (with a scaling factor of 0.947[58]) of $[DKE\text{-}N_3]^-$ (blue), $[DKE\text{-}N_9]^-$ (red), and their combinations (16, 22, and 27% minor tautomer) demonstrating the increase of relative peak intensity of 1578 $cm^{-1}$ to 1555 $cm^{-1}$ and the red shift of C=O bands with increasing $[DKE\text{-}N_9]^-$ population. These DFT calculations mimic experimental trends in Fig. 3d. (**b**) (Top) Animation snapshots showing major difference in IR asymmetric stretching modes of N-H groups between $[DKE\text{-}N_3]^-$ and $[DKE\text{-}N_9]^-$ tautomers. Experimental variable temperature IR spectra (bottom left) of a $D_2O$ solution (pH 7 and 18 mM urate) in the N-H stretching region showing at least four N-H peaks in contrast with simulated IR spectra (bottom right) of $[DKE\text{-}N_3]^-$ (blue) and $[DKE\text{-}N_9]^-$ (red) tautomers displaying only three N-H peaks. The simulated IR spectra of a combination of $[DKE\text{-}N_3]^-$ and $[DKE\text{-}N_9]^-$ with given $[DKE\text{-}N_9]^-$ population mimic the experimental data, consistent with the collective evidence of $[DKE\text{-}N_9]^-$ being a minor tautomer in solution.

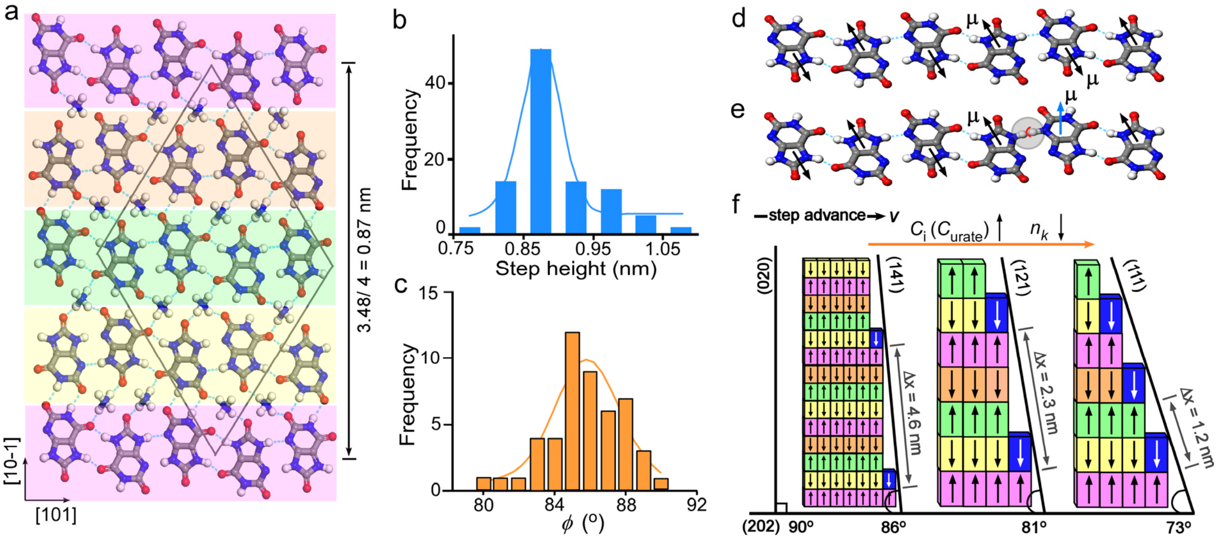


**Supplementary Fig. 12. Inhibitor-crystal interactions and putative mechanism of $NH_4HU$ tapering.** (**a**) Molecular packing in crystal structure, viewed in the b-direction showing four molecules within a lattice spanning 3.48 nm along the [10$\bar{1}$] direction. (**b**) The distribution of step heights on a (20$\bar{2}$) basal surface, measured from *in situ* AFM images of more than 80 steps. The blue line represents a Gaussian fit. The average step height of single layers, 0.9 ± 0.1 nm, corresponds to a single urate molecule. (**c**) Distribution of tapering angle, ϕ=[uvw]∩[101], listed in Fig. 4j, measured from SEM images of more than 50 crystals. The tapering angles span from 78 to 90° with an average value of 86 ± 1°. The yellow line is a Gaussian fit. (**d** and **e**) Comparison of the molecular packing in $NH_4HU$ (d) compared to the incorporation of a single $[DKE\text{-}N_9]^-$ molecule (e) which leads to a repulsion (steric hindrance) of amine groups (grey shaded region) between $[DKE\text{-}N_3]^-$ solute and $[DKE\text{-}N_9]^-$ inhibitor and change of dipole moment (μ, black to blue arrow) arrangement of urate molecules. (**f**) Illustration of different coverage of $[DKE\text{-}N_9]^-$ inhibitor on (202)∩(020) kink sites. The $[DKE\text{-}N_9]^-$ inhibitor putatively binds to kinks in a similar way as $[DKE\text{-}N_3]^-$ solute, through $R_2^2(10)$ hydrogen-bonding interactions at the (202) terrace and π···π stacking interactions at the (020) step. This binding breaks $C_1^1(4)$ hydrogen-bonding chain interactions and creates strong amine-amine repulsion with adjacent $[DKE\text{-}N_3]^-$ dimers (panel e). In the absence of inhibitor, steps advance in the *b* direction with an angle (202)∩(020)=90°; whereas a tapered crystal habit is observed in the presence of $[DKE\text{-}N_9]^-$ with ϕ =(202)∩(hkl) varying between 86° and 73° with increasing surface coverage and low kink density ($n_k$) as the concentration of inhibitor increases. This predicted tapering angle is consistent with SEM images of $NH_4HU$ crystals (Fig. 4j) and AFM measurements (Fig. 2b) of oriented layer advancement on crystal surfaces at pH 7. The high coverage of tautomer adsorption on the step edge will produce an effect of step pinning where the step section stops propagation when gate distance between the pair of tautomer inhibitors, Δx, is smaller than the $2R_c$ whereas partially continues propagation when $\Delta x > 2R_c$ at a given crystallization condition. Such combined effect of kink blocker with step pinner generates a synergistic cooperativity.

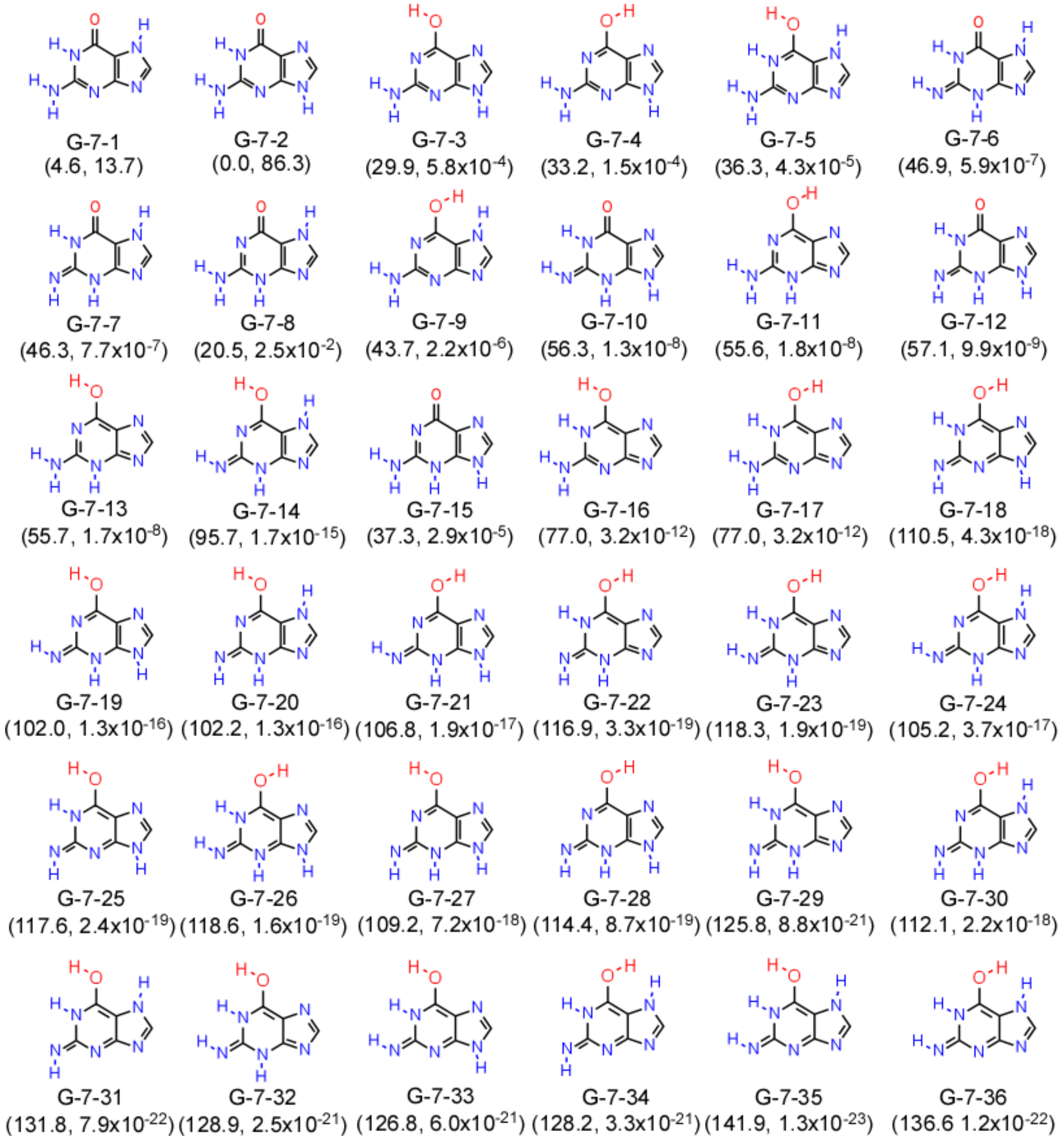


**Supplementary Fig. 13. First principles calculated structures, Gibbs free energy, and population of guanine tautomer species at pH 7 and 25 ºC.** The calculations were performed using CBS-QB3[57] level of theory. The term "G-7-x" represents a guanine (G) tautomer in aqueous solution at pH 7 labeled *x* starting from 1 to 36. The values in parentheses represent relative Gibbs free energy to the most stable tautomer (G-7-2) (kJ/mol) and Boltzmann population (%), respectively. The major tautomer [KA-$N_9$] is G-7-2, and the minor tautomer [KA-$N_7$] is G-7-1.

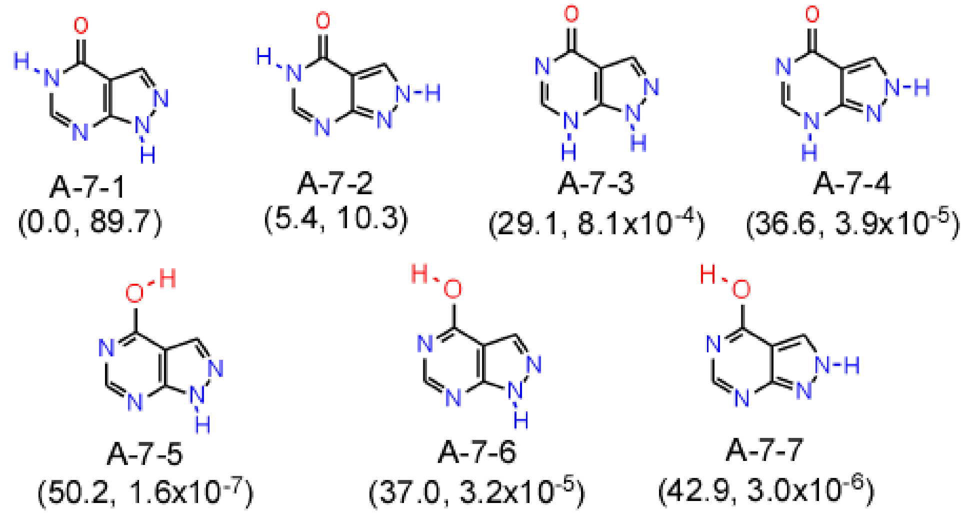


**Supplementary Fig. 14. First principles calculated structures, Gibbs free energy, and population of allopurinol tautomer species at pH 7 and 25 ºC.** The calculations were performed using CBS-QB3[57] level of theory. The term "A-7-x" represents an allopurinol (A) tautomer in aqueous solution at pH 7 labeled *x* starting from 1 to 7. The values in parentheses represent relative Gibbs free energy to the most stable tautomer (A-7-1) (kJ/mol) and Boltzmann population (%), respectively. The major tautomer [K-$N_9$] is A-7-1, and the minor tautomer [K-$N_8$] is A-7-2.

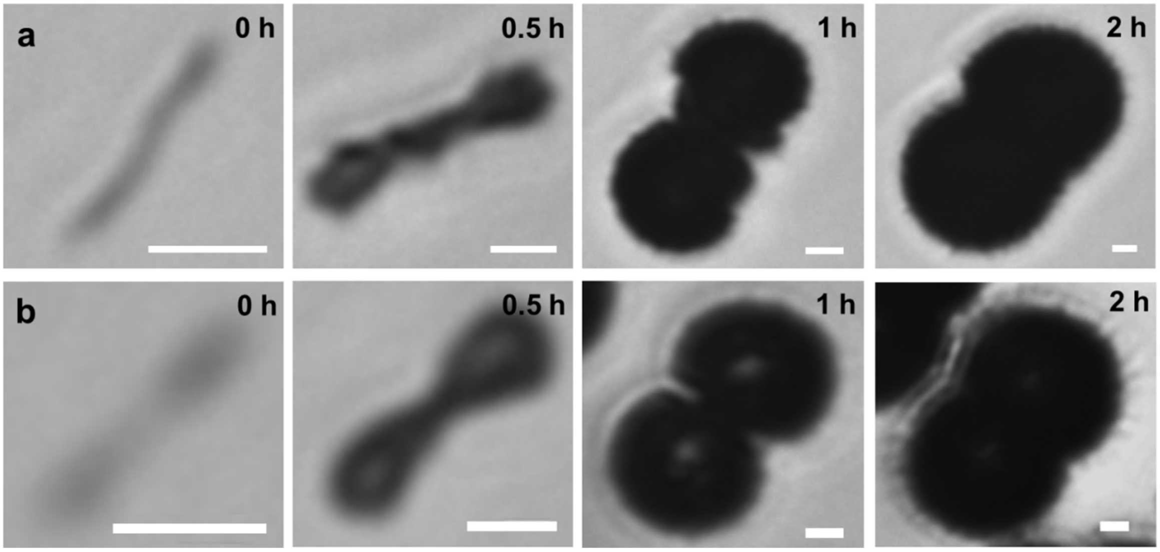


**Supplementary Fig. 15. Effect of NaCl on bulk crystallization.** (**a**) Time-resolved optical micrographs of $NH_4HU$ dendritic growth showing development of dumbbell crystals in 14 mM equimolar sodium urate and ammonium chloride solution (pH 7). (**b**) Time-resolved optical micrographs of $NH_4HU$ dendritic growth showing development of dumbbell crystals in 14 mM equimolar uric acid and ammonium hydroxide solution (pH 7). The experimental results show that crystal morphology in bulk crystallization assays is not affected by the presence of NaCl.

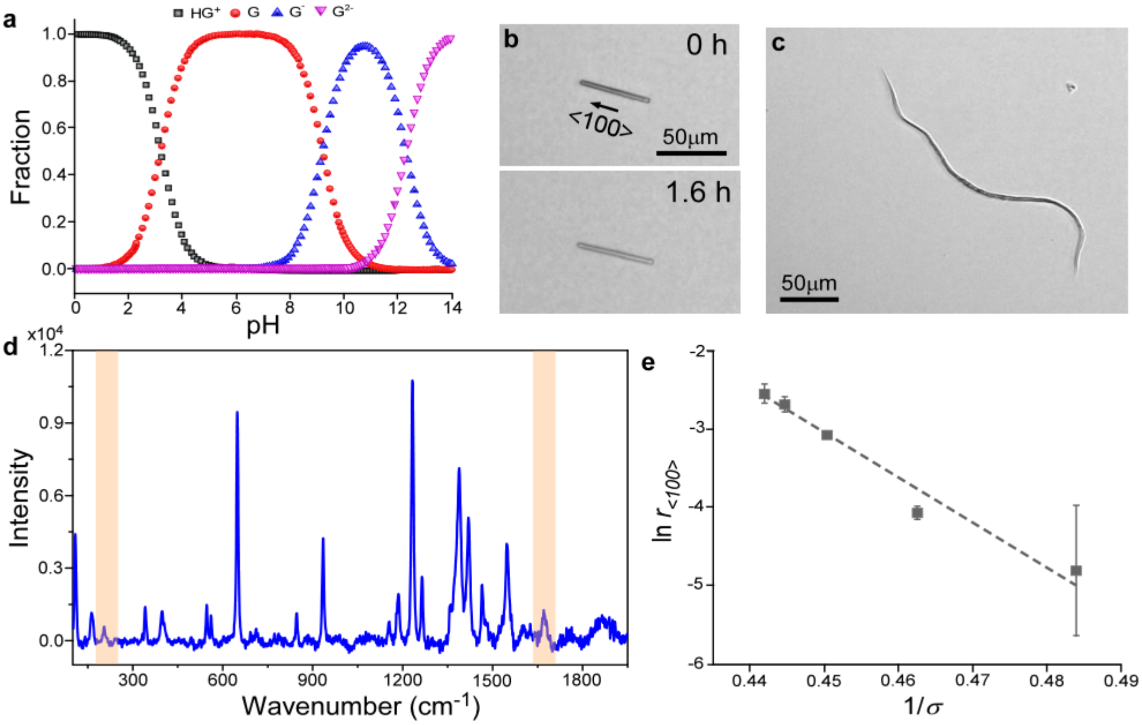


**Supplementary Fig. 16. Effect of pH on guanine crystal growth and morphology.** (**a**) Solution speciation of guanine ($pKa_1$=3.2, $pKa_2$=9.2, and $pKa_3$=12.3) as a function of solution pH: guanine cation ($HG^+$, gray), guanine (G, red), monovalent guanine anion ($G^-$, blue), and divalent guanine anion ($G^{2-}$, purple).[28] (**b**) Time-resolved imaging at lower supersaturation (350 μM guanine at pH 7) showing the slow growth of guanine crystal seeds. The time-elapsed changes in crystal length (<100> direction) during *in situ* microfluidics growth were used to derive the rate of growth reported in Fig. 5f. (**c**) Micrograph image of a bent guanine crystal crystallized in a pH 6 solution for about 2 month. (**d**) Micro-Raman spectrum of a representative guanine seed crystal prepared at pH 7 confirming the β crystal form with two characteristic peaks appeared at 1675 and 204 $cm^{-1}$. The peak at 1675 $cm^{-1}$ corresponding to C=O asymmetry vibration band reveals the anhydrous phase of guanine[28], whereas the unique 204 $cm^{-1}$ peak manifests the β form[59]. (**e**) Linearized plot of growth rates $r_{<100>}$ versus 1/σ reveals an exponential dependence on supersaturation ($r \propto \exp(-B/\sigma)$) where B is the thermodynamic parameter associated with the energy barrier to create a critical radius of 2D islands for the birth and spreading growth mechanism.[50,51]

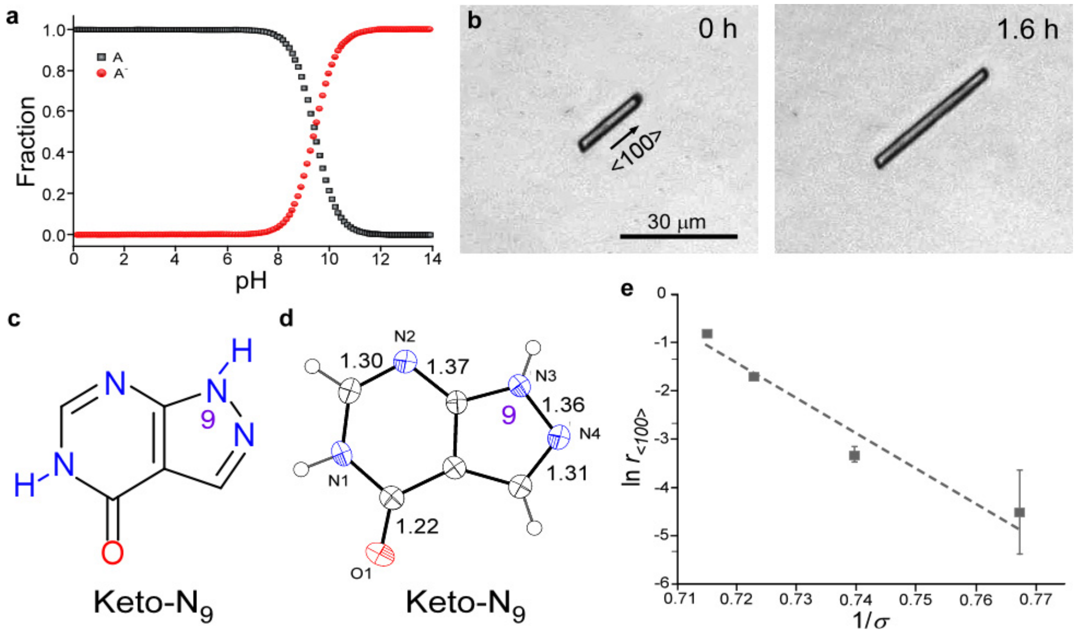


**Supplementary Fig. 17. Effect of pH on allopurinol crystal growth and morphology.** (**a**) Solution speciation of allopurinol (pKa=9.4) as a function of solution pH: allopurinol (A, grey) and monovalent allopurinol anion ($A^-$, red).[29] (**b**) Time-resolved imaging at lower supersaturation (6.5 mM allopurinol at pH 7) showing the fast growth of allopurinol crystal seeds. The time-elapsed changes in crystal length (<100> direction) during *in situ* microfluidics growth were used to derive the rate of growth reported in Fig. 5g and panel e. (**c**) Molecular structure of allopurinol keto-$N_9$ tautomer. (**d**) Geometric bond length (in Angstroms) of allopurinol keto-$N_9$ tautomer within the crystal structure of seed crystals obtained from single-crystal X-ray diffraction. Notably, the H-C-N bond length of the N atom without H attached (1.303(2) Å and 1.313(2) Å) is shorter than others (1.343(2)-1.399(2) Å), which confirms -C=N bond and the positions of hydrogen. (**e**) Linearized plot of growth rates $r_{<100>}$ versus 1/σ reveals an exponential dependence on supersaturation ($r \propto \exp(-B/\sigma)$) where B is the thermodynamic parameter associated with the energy barrier to create a critical radius of 2D islands for the birth and spreading growth mechanism.[50,51]

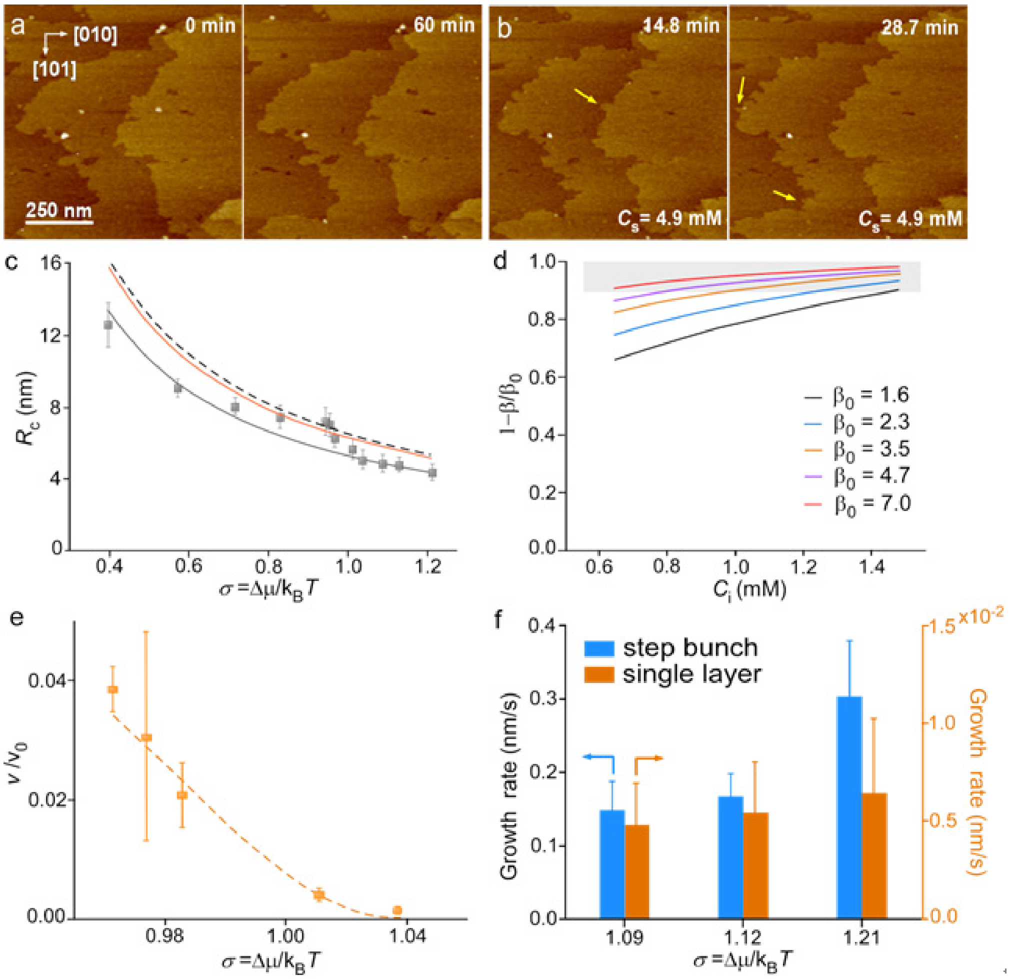


**Supplementary Fig. 18. Inhibitory modes of action for the [DKE-N$_9$]$^-$ tautomer.** (**a**) Time-elapsed *in situ* AFM images showing arrested growth in the "dead zone" (Fig. 2c) at pH 7 and low supersaturation (4.0 mM urate). (**b**) Time-resolved *in situ* AFM images of the region in panel A after increasing the supersaturation (5 mM urate). Numerous step protrusions (yellow arrows) are observed with increasing imaging time. (**c**) Dependence of the critical radius of the two-dimensional nucleus $R_c$ on solution supersaturation $\sigma=\Delta\mu/k_BT$ at pH 7. Symbols are the average of 25 to 35 measurements and error bars span two standard deviations. Solid lines are model predictions using the Gibbs–Thomson relation $R_c = \Omega\alpha/\Delta\mu$ with step line tension α (mJ m$^{-2}$) set equal to 130 (grey line), 153 (yellow line), and 157 (dashed line). The latter was predicted from the Turnbull empirical rule[60] where $\alpha = 0.5\left|\Delta H^0_{cryst}\right|/\Omega^{2/3}$, and $\Delta H^0_{cryst} = -58.2$ kJ mol$^{-1}$ is the crystallization enthalpy determined from the temperature dependence of urate solubility data[26]. (**d**) Derived surface coverage of inhibitor (1-β/β$_0$) on (020) steps with the fitted parameters $A_1$ and $K_i$ from the normalized $v/v_0$ plot (Fig. 4c) prior to growth cessation using Equation (33). Here β and β$_0$ are the kinetic coefficients in the presence and absence of impurity, respectively, and $C_i$ is the concentration of [DKE-N$_9$]$^-$ inhibitor. The shaded grey region indicates 90% coverage. Sensitivity analysis of $\beta_0$ (or $v_0$) suggests high coverage of adsorbed inhibitor before growth cessation (Note: the model fit in Fig. 4c is based on $\beta_0$ = 3.5). (**e**) The steep decline in normalized $v/v_0$ with increasing supersaturation in the growth cessation region (pH 7) can be fit with a kink-limited growth model, $v/v_0 = 1+b_1C_i-b_2C_i^2$, with fitting parameters $b_1$ = -1.0 and $b_2$ = -0.24. Data were taken from Fig. 2c. (**f**) Comparison of growth rates for step bunches (blue) and single layers (orange) from *in situ* AFM in growth solutions at pH 7 and supersaturations σ. Symbols are the average of 5 – 15 measurements and error bars span two standard deviations.

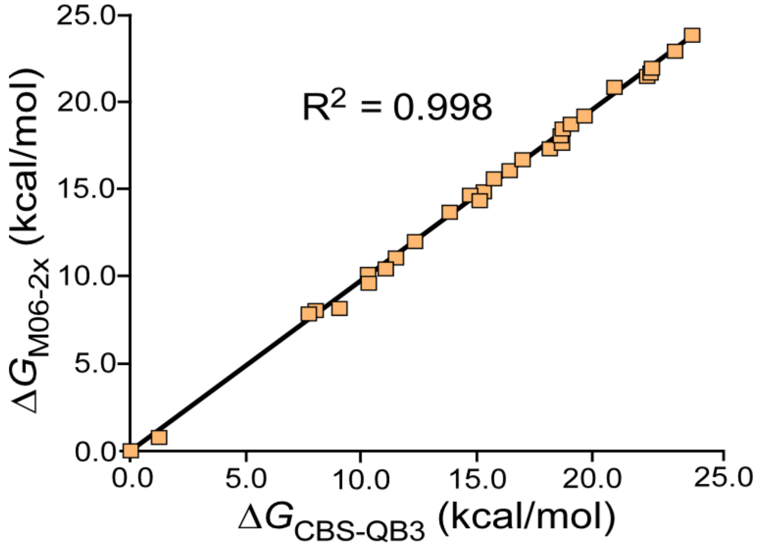


**Supplementary Fig. 19. Computational accuracy of M06-2X.** Free energy comparison of urate monovalent tautomer stability at pH 7 calculated by M06-2X and CBS-QB3 methods (0 is set to the most stable, primary tautomer). The excellent agreement between the two methods demonstrates that the M06-2X method used for UV-Vis simulations is very accurate for the temperature-dependent analysis of the population distribution and its effect on the absorption behavior.

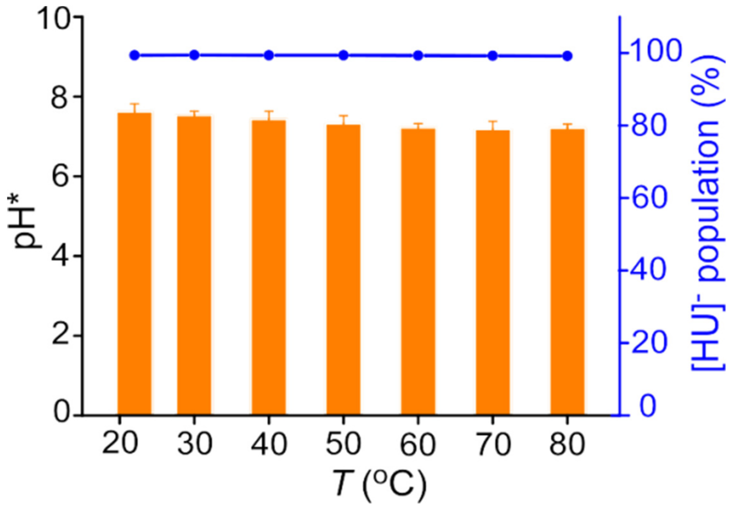


**Supplementary Fig. 20. Variation of urate solution pH* in $D_2O$ at different temperatures throughout variable-temperature IR measurements.** Solution pH* of 18 mM urate in $D_2O$ varies within ±0.2 as temperature varies, and the population of urate monovalent species is all above 99.0%. This reveals the changes in IR spectra are attributed to urate tautomerism and not urate ionization.

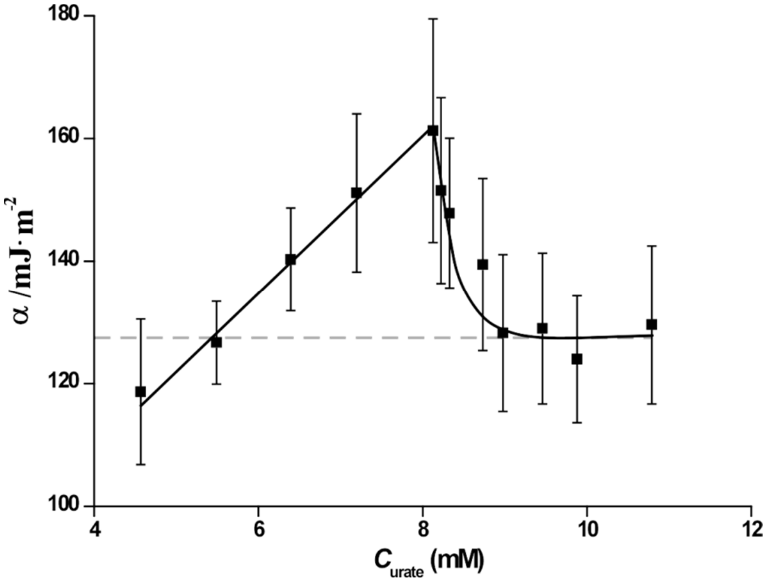


**Supplementary Fig. 21. Calculated surface free energy of step edges with respect to urate concentration at pH 7.** The calculation was performed from measured critical size of 2D islands (Supplementary Fig. 18c), according to Gibbs–Thomson relation $\alpha = \Delta\mu R_c/\Omega$. Error bars represent two standard deviations from the average of 25 to 35 $R_c$ measurements.

## 4. Supplementary Tables

**Supplementary Table 1.** Experimental parameters for 3D ED data collection and crystallographic data for $NH_4HU$ refinement using combined 3D ED and Pawley fitting.

| | 3D ED * |
|---|---|
| **Number of datasets merged** | 10 |
| **Completeness (%)** | 96.7 |
| **Rotation speed (/s)** | 0.6 |
| **Exposure time (s/frame)** | 1.0 |
| **Formula** | $C_5H_7N_5O_3$ |
| **Formula weight** | 185.14 |
| **Temperature (K)** | 293(2) |
| **Crystal system** | Monoclinic |
| **Space group (no.)** | *C*2/c (15) |
| ***a*** **(Å)** | 21.385(4) |
| ***b*** **(Å)** | 3.5300(7) |
| ***c*** **(Å)** | 20.080(4) |
| **α (º)** | 90 |
| **β (º)** | 114.10(3) |
| **γ (º)** | 90 |
| ***V*** **($Å^3$)** | 1383.7(6) |
| ***Z*** | 8 |
| **$D_c$ (g $cm^{-3}$)** | 1.789 |
| ***F*****(000)** | 259.0 |
| **Radiation** | 0.0197 |
| **Resolution range (Å)** | 10.69 to 0.85 |
| **Index range** | $-24 \le h \le 24$,<br>$-4 \le k \le 4$<br>$-23 \le l \le 23$ |
| **Reflections collected** | 26722 |
| **$R_{int}$** | 0.2635 |
| **Data/restraints/parameters** | 1239/15/147 |
| **GOF on $F^2$** | 1.165 |
| **Final R indexes [$F_o > 4sig(F_o)$]** | $R_1$=0. 1928 |
| **Final R indexes [all data]** | $R_1$= 0. 2106 |

*Structures (.cif files) from 3D ED analysis and Rietveld refinement were deposited in the CCDC (2110687 and 2121918, respectively)

**Supplementary Table 2.** Elemental Analyses of C/H/N Ratio in $NH_4HU$ Crystal Samples.*

| | C | H | N |
|---|---|---|---|
| Run 1[a] | 31.77±0.03% | 3.73±0.17% | 36.38±0.01% |
| Run 2[b] | 31.99±0.01% | 3.64±0.03% | 36.93±0.05% |
| Run 3[c] | 32.49±0.01% | 3.58±0.01% | 37.65±0.12% |
| $NH_4HU$ | 32.43% (-0.43%) | 3.78% (-0.14%) | 37.84% (-0.84%) |
| $NH_4HU·NH_3$ | 29.70% (-2.3%) | 4.95% (+1.31%) | 41.58% (+4.58%) |
| $NH_4HU·H_2O$ | 29.56% (-2.44%) | 4.43% (+0.79%) | 34.48% (+2.52%) |

[a] Solution formula: 14 mM sodium urate with ammonium chloride; [b] Solution formula: 14 mM uric acid with ammonium hydroxide at pH 7; [c] Solution formula: 10mM uric acid with ammonium hydroxide at pH 11; * The value in parentheses represents absolute standard deviation.

**Supplementary Table 3.** Rate constant $k$ and half life time $t_{1/2}$ for interconversion reaction kinetics between diketo-enol tautomers (i.e. $[DKE\text{-}N_3]^-$ and $[DKE\text{-}N_9]^-$) at different temperatures.

| **Temp (°C)** | $\mathbf{k_1}$ **($M^{-1}\cdot s^{-1}$)** | $\mathbf{k_2}$ **($M^{-1}\cdot s^{-1}$)** | $\mathbf{k}$ **($s^{-1}$)** | $\mathbf{t_{1/2}}$ **(s)** |
|---|---|---|---|---|
| 5 | 0.1 | 0.4 | 32.5 | $2.1x10^{-2}$ |
| 15 | 0.4 | 1.1 | 81.5 | $8.5x10^{-3}$ |
| 25 | 0.9 | 2.5 | 192.4 | $3.6x10^{-3}$ |
| 35 | 2.1 | 4.8 | 382.9 | $1.8x10^{-3}$ |
| 45 | 4.6 | 10.1 | 818.3 | $8.5x10^{-4}$ |
| 55 | 11.1 | 23.9 | 1948.4 | $3.6x10^{-4}$ |
| 65 | 22.0 | 46.4 | 3801.9 | $1.8x10^{-4}$ |
| 70 | 28.4 | 51.1 | 4417.2 | $1.6x10^{-4}$ |

**Supplementary Table 4.** Crystallographic data of allopurinol anhydrous form reported in this study in comparison with the literature[61].

| | Allopurinol (this study) | Allopurinol (literature[61]) |
|---|---|---|
| **Formula** | $C_5H_4N_4O$ | $C_5H_4N_4O$ |
| **Formula weight** | 136.12 | 136.12 |
| **Temperature/K** | 294 | / |
| **wavelength** | 0.71073 | 1.5418 |
| **Crystal system** | monoclinic | monoclinic |
| **Space group** | $P\ 2_1/c$ | $P\ 2_1/c$ |
| **a/ Å** | 3.6752(5) | 3.683(1) |
| **b/ Å** | 14.6886(14) | 14.685(3) |
| **c/ Å** | 10.3201(11) | 10.318 (2) |
| **α/°** | 90 | 90 |
| **β/°** | 97.498(12) | 97.47(2) |
| **γ/°** | *90* | *90* |
| **V/Å$^{-3}$** | 552.35(11) | 553.31 |
| **$\rho_{calc}$/g cm$^{-3}$** | 1.637 | 1.635 |
| **Z, Z'** | 4, 1 | 4, 1 |
| **$R_{int}$** | 0.0881 | / |
| **R(ref)** | 0.0459 | 0.045 |
| **$wR_2$(ref)** | 0.1194 | / |
| **CCDC** | 2206318 | 1102293 |

**Supplementary Table 5.** Fitted values of adsorption constant $K_i$, kink density parameter $A_1$, edge free energy parameter $A_2$ from Equation 34 in a series of $\beta_0$ values.

| $\beta_0$ (μm· s$^{-1}$) | **$A_1$ (-)** | **$K_i$ (mM$^{-1}$)** | **$A_2$ (-)** |
|---|---|---|---|
| 7.0 | 1.0 | 4.8 | 0.36 |
| 4.7 | 1.1 | 2.9 | 0.38 |
| 3.5 | 1.1 | 1.9 | 0.41 |
| 2.3 | 1.2 | 0.90 | 0.49 |
| 1.6 | 1.6 | 0.34 | 0.69 |

## 5. Supplementary References


1 Yang, T., Xu, H. & Zou, X. Improving data quality for 3D electron diffraction (3D ED) by Gatan Image Filter and a new crystal tracking method. *arXiv preprint arXiv:2108.07468* (2021).

2 Wan, W., Sun, J., Su, J., Hovmoller, S. & Zou, X. Three-dimensional rotation electron diffraction: software RED for automated data collection and data processing. *J. Appl. Crystallogr.* **46**, 1863-1873 (2013).

3 Kabsch, W. XDS. *Acta Crystallogr., Sect D: Biol. Crystallogr.* **66**, 125-132 (2010).

4 Wang, B., Zou, X. & Smeets, S. Automated serial rotation electron diffraction combined with cluster analysis: an efficient multi-crystal workflow for structure determination. *IUCrJ* **6**, 854-867 (2019).

5 Sheldrick, G. SHELXT - Integrated space-group and crystal-structure determination. *Acta Crystallogr., Set. A: Found. Crystallogr.* **71**, 3-8 (2015).

6 Sheldrick, G. Crystal structure refinement with SHELXL. *Acta Crystallogr., Sect. C: Cryst. Struct. Commun.* **71**, 3-8 (2015).

7 Boultif, A. & Louer, D. Powder pattern indexing with the dichotomy method. *J. Appl. Crystallogr.* **37**, 724-731 (2004).

8 David, W. I. F. *et al.* DASH: a program for crystal structure determination from powder diffraction data. *J. Appl. Crystallogr.* **39**, 910-915 (2006).

9 Coelho, A. Indexing of powder diffraction patterns by iterative use of singular value decomposition. *J. Appl. Crystallogr.* **36**, 86-95 (2003).

10 Coelho, A. TOPAS and TOPAS-Academic: an optimization program integrating computer algebra and crystallographic objects written in C++. *J. Appl. Crystallogr.* **51**, 210-218 (2018).

11 Pawley, G. Unit-cell refinement from powder diffraction scans. *J. Appl. Crystallogr.* **14**, 357-361 (1981).

12 David, W. I. F. & Shankland, K. Structure determination from powder diffraction data. *Acta Crystallogr., Set. A: Found. Crystallogr.* **64**, 52-64 (2008).

13 Rietveld, H. Line profiles of neutron powder-diffraction peaks for structure refinement. *Acta Crystallogr.* **22**, 151-152 (1967).

14 Rietveld, H. A profile refinement method for nuclear and magnetic structures. *J. Appl. Crystallogr.* **2**, 65-71 (1969).

15 Rasband, W. S. Imagej, us national institutes of health, bethesda, maryland, usa. *http://imagej. nih. gov/ij/* (2011).

16 Kisielowski, C. *et al.* Modulating Electron Beam–Sample Interactions in Imaging and Diffraction Modes by Dose Fractionation with Low Dose Rates. *Microsc. Microanal.*, 1-11 (2021).

17 *https://www.totalresolution.com/*.

18 Zauscher, S., Parlak, Z. & Tu, Q. in *Handbook of nanomaterials properties* 1023-1051 (Springer, 2014).

19 Rabe, U. in *Applied scanning probe methods II* 37-90 (Springer, 2006).

20 Come, J. *et al.* Nanoscale elastic changes in 2D Ti3C2Tx (MXene) pseudocapacitive electrodes. *Advanced Energy Materials* **6**, 1502290 (2016).

21 Rabe, U. *et al.* Quantitative determination of contact stiffness using atomic force acoustic microscopy. *Ultrasonics* **38**, 430-437 (2000).

22 Killgore, J. P. & Hurley, D. C. Low-force AFM nanomechanics with higher-eigenmode contact resonance spectroscopy. *Nanotechnology* **23**, 055702 (2012).

23 Dolbow, J. & Gosz, M. Effect of out-of-plane properties of a polyimide film on the stress fields in

microelectronic structures. *Mech. Mater.* **23**, 311-321 (1996).

24 Evans, M. G. & Polanyi, M. Some applications of the transition state method to the calculation of reaction velocities, especially in solution. *Trans. Faraday Soc.* **31**, 875-894 (1935).

25 Kahn, K., Serfozo, P. & Tipton, P. A. Identification of the True Product of the Urate Oxidase Reaction. *J. Am. Chem. Soc.* **119**, 5435-5442 (1997).

26 Geng, X., Meegan, J., Smith, C., Sakhaee, K. & Rimer, J. D. Crystallization of Hierarchical Ammonium Urate: Insight into the Formation of Cetacean Renal Stones. *Cryst. Growth Des.* **19**, 6727-6735 (2019).

27 Stumm, W. & Morgan, J. J. *Aquatic chemistry: chemical equilibria and rates in natural waters*. 3 edn, Vol. 126 (John Wiley & Sons, 2012).

28 Gur, D. *et al.* Guanine Crystallization in Aqueous Solutions Enables Control over Crystal Size and Polymorphism. *Cryst. Growth Des.* **16**, 4975-4980 (2016).

29 Zhou, C. *et al.* Rapid pKa estimation using vacuum-assisted multiplexed capillary electrophoresis (VAMCE) with ultraviolet detection. *J. Pharm. Sci.* **94**, 576-589 (2005).

30 Cabrera, N. V., D. A. *Growth of Crystals from Solution (eds Doremus R. H. et al.) Ch. V, 393-410*. 393-410 (Wiley, 1958).

31 Voronkov, V. V. & Rashkovich, L. N. Step kinetics in the presence of mobile adsorbed impurity. *J. Cryst. Growth* **144**, 107-115 (1994).

32 Weaver, M. L. *et al.* Improved Model for Inhibition of Pathological Mineralization Based on Citrate–Calcium Oxalate Monohydrate Interaction. *ChemPhysChem* **7**, 2081-2084 (2006).

33 Weaver, M. L. *et al.* Inhibition of calcium oxalate monohydrate growth by citrate and the effect of the background electrolyte. *J. Cryst. Growth* **306**, 135-145 (2007).

34 Chernov, A. A. *Modern crystallography III: crystal growth*. Vol. 36 (Springer Science & Business Media, 2012).

35 Chernov, A. A. The spiral growth of crystals. *Sov. Phys. Usp.* **4**, 116-148 (1961).

36 Ma, W., Lutsko, J. F., Rimer, J. D. & Vekilov, P. G. Antagonistic cooperativity between crystal growth modifiers. *Nature* **577**, 497-501 (2020).

37 Davey, R. J. The effect of impurity adsorption on the kinetics of crystal growth from solution. *J. Cryst. Growth* **34**, 109-119 (1976).

38 Bliznakov, B. *Fortschr. Mineral.* , 149-191 (1958).

39 Sleutel, M. & Van Driessche, A. E. S. On the Self-Purification Cascade during Crystal Growth from Solution. *Cryst. Growth Des.* **13**, 688-695 (2013).

40 Olafson, K. N., Nguyen, T. Q., Rimer, J. D. & Vekilov, P. G. Antimalarials inhibit hematin crystallization by unique drug-surface site interactions. *Proc. Nat. Acad. Sci. USA* **114**, 7531-7536 (2017).

41 Petersen, P. B. & Saykally, R. J. ON THE NATURE OF IONS AT THE LIQUID WATER SURFACE. *Annu. Rev. Phys. Chem.* **57**, 333-364 (2006).

42 Hey, M. J., Shield, D. W., Speight, J. M. & Will, M. C. Surface tensions of aqueous solutions of some 1:1 electrolytes. *J. Chem. Soc., Faraday Trans. 1* **77**, 123-128 (1981).

43 Adamsom, A. & Gast, A. Physical chemistry of surfaces. *A Wiley-Interscience Publication* (1997).

44 Durand-Vidal, S., Simonin, J.-P. & Turq, P. *Electrolytes at interfaces*. Vol. 1 (Springer Science & Business Media, 2001).

45 Gibbs, J. W. On the Equilibrium of Heterogeneous Substances. *Trans. Connect. Acad. Sci.* **3**, 108–248 (1876).

46 Yoreo, J. J. D. & Vekilov, P. G. Principles of Crystal Nucleation and Growth. *Rev. Mineral.*

*Geochem.* **54**, 57-93 (2003).

47 De Yoreo, J. J. *et al.* Rethinking Classical Crystal Growth Models through Molecular Scale Insights: Consequences of Kink-Limited Kinetics. *Cryst. Growth Des.* **9**, 5135-5144 (2009).

48 Chen, J. *et al.* Building two-dimensional materials one row at a time: Avoiding the nucleation barrier. *Science* **362**, 1135-1139 (2018).

49 Friedel, P., Bergmann, J., Kleeberg, R. & Schubert, G. A proposition for the structure of ammonium hydrogen (acid) urate from uroliths. *Z. Kristallogr.* **2**, 522 (2006).

50 Davey, R. & Garside, J. *From molecules to crystallizers*. (Oxford University Press, 2000).

51 Calvert, P. D., Fiddis, R. W. & Vlachos, N. Crystal growth of monosodium urate monohydrate. *Colloids Surf.* **14**, 97-107 (1985).

52 Zhao, Y. & Truhlar, D. G. The M06 suite of density functionals for main group thermochemistry, thermochemical kinetics, noncovalent interactions, excited states, and transition elements: two new functionals and systematic testing of four M06-class functionals and 12 other functionals. *Theor. Chem. Acc.* **120**, 215-241 (2008).

53 Zhao, Y. & Truhlar, D. G. A new local density functional for main-group thermochemistry, transition metal bonding, thermochemical kinetics, and noncovalent interactions. *J. Chem. Phys.* **125**, 194101 (2006).

54 Gilmer, G. H., Ghez, R. & Cabrera, N. An analysis of combined surface and volume diffusion processes in crystal growth. *J. Cryst. Growth* **8**, 79-93 (1971).

55 Vekilov, P. G., Kuznetsov, Y. G. & Chernov, A. A. The effect of temperature on step motion; (101) ADP face. *J. Cryst. Growth* **121**, 44-52 (1992).

56 Olafson, K. N., Ketchum, M. A., Rimer, J. D. & Vekilov, P. G. Molecular Mechanisms of Hematin Crystallization from Organic Solvent. *Cryst. Growth Des.* **15**, 5535-5542 (2015).

57 Jr., J. A. M., Frisch, M. J., Ochterski, J. W. & Petersson, G. A. A complete basis set model chemistry. VI. Use of density functional geometries and frequencies. *J. Chem. Phys.* **110**, 2822-2827 (1999).

58 Kashinski, D. O. *et al.* Harmonic Vibrational Frequencies: Approximate Global Scaling Factors for TPSS, M06, and M11 Functional Families Using Several Common Basis Sets. *J. Phys. Chem. A* **121**, 2265-2273 (2017).

59 Jantschke, A. *et al.* Anhydrous β-guanine crystals in a marine dinoflagellate: Structure and suggested function. *J. Struct. Biol.* **207**, 12-20 (2019).

60 Turnbull, D. Formation of Crystal Nuclei in Liquid Metals. *J. Appl. Phys.* **21**, 1022-1028 (1950).

61 Prusiner, P. & Sundaralingam, M. Stereochemistry of nucleic acids and their constituents. XXIX. Crystal and molecular structure of allopurinol, a potent inhibitor of xanthine oxidase. *Acta Crystallogr. B* **28**, 2148-2152 (1972).
