# Supplementary material for: Tautomerism unveils a self-inhibition mechanism of crystallization
Source: Nat Commun. 2023 Feb 2;14:561. doi: 10.1038/s41467-023-35924-3 (PMC9893984; doi:10.1038/s41467-023-35924-3)
Supplement: Supplementary file 3 — Description of Additional Supplementary Files [file 41467_2023_35924_MOESM3_ESM.pdf]

Description of Additional Supplementary Files

File Name: Supplementary Movie 1
Description: 3D reciprocal space reconstructed from a tilt series of electron diffraction patterns of a NH4HU crystal prepared at pH 11 viewing from different angles.

File Name: Supplementary Movie 2
Description: Time-elapsed in situ AFM images for data in Fig. 2A showing NH4HU surface growth in a supersaturated solution (8.1 mM urate, pH 7), selected within the regime of crystal bending, where continuous scanning in tapping mode reveals the birth and spreading of 2D islands.

File Name: Supplementary Movie 3
Description: Time-elapsed in situ AFM images for data in Fig. 2D showing NH_4HU surface growth in a supersaturated solution (12 mM urate, pH 7) where continuous scanning in tapping mode reveals the evolution of multilayer step bunches that advanced in ±b directions. The first 9 min of the video corresponds to the period of substrate equilibration at conditions corresponding to the dead zone (4 mM urate, pH 7) prior to the addition of the growth solution.

File Name: Supplementary Movie 4
Description: Time-elapsed in situ AFM images for data in Fig. 2E showing NH_4HU surface growth in a supersaturated solution (9.5 mM urate, pH 7) where continuous scanning in tapping mode reveals the onset of step bunching.

File Name: Supplementary Movie 5
Description: Time-elapsed in situ AFM images for data in Fig. 2G showing NH4HU surface growth in a supersaturated solution (12 mM urate, pH 11) where continuous scanning in tapping mode reveals classical layer-bylayer growth.

File Name: Supplementary Movie 6
Description: Animation showing the inter-conversion between tautomers $[DKE\text{-}N_9]^-$ and $[DKE\text{-}N_3]^-$ in neutral solution based on DFT calculations. The interconversion, obtained from the IRC calculation, involves a watermediated hydrogen transfer and is shown starting from $[DKE\text{-}N_9]^-$ and ending to $[DKE\text{-}N3]^-$.

File Name: Supplementary Movie 7
Description: Time-elapsed in situ optical micrographs showing dissolution of NH4HU crystals prepared at pH 7 in a microfluidic device. Continuous imaging in DI water at a continuous flow rate of 6 mL h^{-1} reveals inhomogeneous dissolution over an 18-min period.

File Name: Supplementary Movie 8
Description: Time-elapsed in situ optical micrographs showing dissolution of NH4HU crystals prepared at pH 11 in a microfluidic device. Continuous imaging in DI water at a continuous flow rate of 6 mL h^{-1} reveals homogeneous dissolution over a 36-min period.
